# Novel prokaryotic expression of thioredoxin-fused insulinoma associated protein tyrosine phosphatase 2 (IA-2), its characterization and immunodiagnostic application

**DOI:** 10.1186/s12896-016-0309-2

**Published:** 2016-11-24

**Authors:** Luciano Lucas Guerra, Natalia Inés Faccinetti, Aldana Trabucchi, Bruno David Rovitto, Adriana Victoria Sabljic, Edgardo Poskus, Ruben Francisco Iacono, Silvina Noemí Valdez

**Affiliations:** Universidad de Buenos Aires, Consejo Nacional de Investigaciones Científicas y Técnicas, Instituto de Estudios de la Inmunidad Humoral “Prof. Ricardo A. Margni” (IDEHU), Facultad de Farmacia y Bioquímica, Buenos Aires, Argentina

**Keywords:** IA-2, Recombinant protein expression, Diabetes Mellitus, Autoantibody, Autoimmunity, Immunoassay, *Escherichia coli*

## Abstract

**Background:**

The insulinoma associated protein tyrosine phosphatase 2 (IA-2) is one of the immunodominant autoantigens involved in the autoimmune attack to the beta-cell in Type 1 Diabetes Mellitus. In this work we have developed a complete and original process for the production and recovery of the properly folded intracellular domain of IA-2 fused to thioredoxin (TrxIA-2_ic_) in *Escherichia coli* GI698 and GI724 strains. We have also carried out the biochemical and immunochemical characterization of TrxIA-2_ic_and design variants of non-radiometric immunoassays for the efficient detection of IA-2 autoantibodies (IA-2A).

**Results:**

The main findings can be summarized in the following statements: i) TrxIA-2_ic_ expression after 3 h of induction on GI724 strain yielded ≈ 10 mg of highly pure TrxIA-2_ic_/L of culture medium by a single step purification by affinity chromatography, ii) the molecular weight of TrxIA-2_ic_ (55,358 Da) could be estimated by SDS-PAGE, size exclusion chromatography and mass spectrometry, iii) TrxIA-2_ic_ was properly identified by western blot and mass spectrometric analysis of proteolytic digestions (63.25 % total coverage), iv) excellent immunochemical behavior of properly folded full TrxIA-2_ic_ was legitimized by inhibition or displacement of [^35^S]IA-2 binding from IA-2A present in Argentinian Type 1 Diabetic patients, v) great stability over time was found under proper storage conditions and vi) low cost and environmentally harmless ELISA methods for IA-2A assessment were developed, with colorimetric or chemiluminescent detection.

**Conclusions:**

*E. coli* GI724 strain emerged as a handy source of recombinant IA-2_ic_, achieving high levels of expression as a thioredoxin fusion protein, adequately validated and applicable to the development of innovative and cost-effective immunoassays for IA-2A detection in most laboratories.

**Electronic supplementary material:**

The online version of this article (doi:10.1186/s12896-016-0309-2) contains supplementary material, which is available to authorized users.

## Background

The insulinoma associated protein tyrosine phosphatase 2 (IA-2) is a 106 kDa (979 residues) transmembrane glycoprotein [1–3] whose encoding gene is localized in human chromosome 2q35. IA-2 is expressed in neuroendocrine cells, such as the peptidergic neurons of the central nervous system, the chromaffin cells present in the adrenal medulla and the endocrine pancreas (alpha and beta-cells of the pancreatic islet) [3–5]. IA-2 belongs to the protein tyrosine phosphatase (PTP) family, particularly to the receptor type PTPs; however, it lacks such enzymatic activity [6, 7]. In pancreatic islets, IA-2 participates in the cytoplasmic transport of dense core secretory granules (DSG) containing insulin, its subsequent excretion into the extracellular space, biogenesis, storage and recycling, as well as in beta-cell proliferation [8–12]. The IA-2 structure consists of three domains: the extracellular domain (residues 1–576, which are in contact with the DSG lumen during storage) that is separated from the intracellular domain or IA-2_ic_ (residues 601–979, oriented to the beta-cell cytoplasm) by a single transmembrane domain (residues 577–600). It was demonstrated that, after exocytosis, calpain mediates the release of IA-2_ic _from the plasma membrane; a process after which IA-2 travels to the nucleus where its inactive PTP domain binds to the phosphorylated tyrosine present in the STAT5 protein, thus upregulating gene expression of DSG proteins, including insulin and IA-2 [13–16].

Diabetes Mellitus (DM) is a group of metabolic diseases of heterogeneous etiology characterized by poor metabolic control of patients and the presence of marked hyperglycemia, which stems from defects in insulin secretion and/or action [17]. According to the American Diabetes Association, DM can be classified into four main groups: Type 1, Type 2, gestational and other types. Type 1 DM (T1DM) is characterized by the destruction of pancreatic beta-cells leading to a deficiency in insulin secretion that results in elevated blood glucose levels [18–20]. The loss of tolerance to pancreatic islet antigens is known to be mediated by a T cell-dependent autoimmune process. IA-2 is one of the immunodominant autoantigens involved in the autoimmune attack on the beta-cell in DM. The other autoantigens implicated are: insulin, glutamic acid decarboxylase (GAD) and Zinc transporter 8 (ZnT8). The autoantibodies specific to insulin, GAD, IA-2 and ZnT8 (IAA, GADA, IA-2A and ZnT8A, respectively) can be detected in patient sera as the first detectable sign of emerging beta-cell autoimmunity [21–23]. These autoantibodies are currently used as additional serological markers to confirm the autoimmune process underlying insulin deficiency in diabetic patients. These markers are useful for predicting T1DM in children and to achieve the correct diagnosis in adult patients with intermediate forms of DM, like Latent Autoimmune Diabetes of the Adults (LADA) [24–26].

IA-2A mostly recognize the IA-2_ic_ (essentially the PTP sequence, residues 696–979), since this domain is the one exposed upon cell damage. IA-2A bind to discontinuous epitopes, therefore, the correct folding of IA-2_ic_ is critical for immune recognition [27–30]. Moreover, the diagnostic sensitivity in both newly diagnosed type 1 diabetic patients and pre-diabetic individuals is highest when IA-2_ic_ is used as antigen for IA-2A recognition, thus suggesting that this domain should be used as detection antigen in T1DM–related autoantibody screening studies [31, 32]. Approximately, 60–80 % of patients with T1DM are positive for IA-2A [33, 34]. The probability of T1DM patients’ first-degree relatives contracting DM within 5 years from the detection of the positive marker is 65–85 % for IA-2A (positive predictive value), since these autoantibodies are known to appear later during the development of the autoimmune process. Therefore, the detection of IA-2A can be considered indicative of rapid disease progression [35, 36].

The assessment of a complete set of autoantibodies is not only applied to confirm the existence of an underlying autoimmune process in infant-juvenile or adult diabetic patients (clinical classification of DM), and to monitor first-degree relatives of diabetic individuals (population at risk), but also to predict the need for insulin treatment. Besides, the presence of autoantibodies can be used as inclusion criteria for enrolment in prediction and prevention trials and as an endpoint in observational studies [31, 37–40]. The usefulness of IA-2A detection lies in its high diagnostic specificity, since the frequency of such a marker in healthy individuals is only 2–4 % [31, 41, 42]. The reference method for detection of IA-2A is the Radioligand Binding Assay (RBA) [43], which includes a recombinant radiolabelled autoantigen as tracer for immunocomplex formation in a fluid phase. This tracer is usually synthesized in cell-free eukaryotic systems, bearing [^35^S]-marked aminoacids, making it environmentally inappropriate, expensive and limited to authorized laboratories. The development of more accurate, sensitive and inexpensive methods to detect IA-2A can be helpful to achieve an early diagnosis or prediction of DM. For these reasons, a handy source of properly folded recombinant antigen is needed for the development of innovative and cost-effective non-radiometric immunoassays for the quasi-quantitative assessment of IA-2A (such as solid phase ELISA-type assays) that are applicable to low or medium complexity laboratories [44].

The most widespread recombinant expression system is the prokaryotic one employing *Escherichia coli.* This system has proved to be easy to handle, it is inexpensive and the protein expression yield is high [45, 46]. Different fusion partners are often used to facilitate solubilisation and/or purification of eukaryotic proteins in this system, such as the glutathione-S-transferase, the mannose binding protein or thioredoxin (Trx) [47–49]. We have previously described the expression of IA-2 in *E. coli*; however, the recombinant protein obtained was not stable enough to be implemented in the routine diagnosis of autoimmune DM [50]. In this work we have developed a complete and original process for the production and recovery of the properly folded intracellular domain of IA-2 fused to Trx (TrxIA-2_ic_) in a prokaryotic expression system with high performance. We have also carried out the biochemical and immunochemical characterization of TrxIA-2_ic_ and design variants of non-radiometric immunoassays for the efficient detection of IA-2A using the stable recombinant antigen produced.

## Methods

### Sera collection

#### Argentinian healthy control individuals

Control sera (*n* = 115, mean age of 29.7 years with median age of 25, range 16 to 80 and male/female: 58/57) were obtained from Argentinian healthy individuals without personal or family history of DM or autoimmune diseases. The sample collection was approved by the Ethics Committee of the José de San Martín Clinical Hospital, University of Buenos Aires (UBA), Buenos Aires, Argentina. All subjects were informed about the purpose of the study, and a signed consent for study participation was obtained. Autoantibodies assessment was performed and all of the individuals were negative. Sera were stored at -20 °C until assayed.

#### Argentinian Type 1 diabetic patients

Serum samples (*n* = 60, mean age of 9.2 years with median age of 9, range 2 to 21 and male/female: 23/37) were collected from Argentinian children and adolescents admitted to the Nutrition Service at Gutiérrez National Pediatric Hospital, Buenos Aires, Argentina, from May 2013 to March 2015. Samples from patients fasted overnight (ON) were taken before or within 72 h of starting insulin treatment. T1DM was diagnosed according to WHO criteria [51]. Sample collection and protocols were approved by the Ethics Committees of the Gutiérrez National Pediatric Hospital. Parental consent was obtained. Sera were stored at -20 °C until assayed.

#### Rabbit policlonal sera against IA-2

Recombinant IA-2_ic_ fused to a C-terminal His-tag (IA-2_ic_His_6_) was obtained as previously described by Sica et al [50]. IA-2 antibodies were obtained through immunization of New Zealand white rabbits (*n* = 3) with 0.1 mg of IA-2_ic_His_6_ emulsified in complete Freund’s adjuvant. The initial injection was followed by booster injections with 0.1 mg of IA-2_ic_His_6_ in incomplete Freund’s adjuvant at three-week intervals. Rabbits were bled 15 days after the booster dose.

#### Rabbit polyclonal sera against thioredoxin

Recombinant Trx was expressed in *E. coli* with the pTrx vector (Invitrogen, Carlsbad, CA, USA) and purified by osmotic shock according to the manufacturer’s instructions. The product was dialyzed against phosphate-buffered saline (PBS, 1.5 mM KH_2_PO_4_, 8.1 mM Na_2_HPO_4_, 0.14 M NaCl, 2.7 mM KCl, and pH 7.4) and lyophilized. The antiserum against Trx was obtained by immunizing New Zealand white rabbits (*n* = 3) with 1.0 mg of recombinant Trx emulsified in complete Freund’s adjuvant. The initial injection was followed by booster injections with 1.0 mg of Trx in incomplete Freund’s adjuvant at four-week intervals. Rabbits were bled 15 days after boosting. All animals were housed under specific conditions according to the “Guide for the Care and Use of Laboratory Animals” by the National Research Council of the National Academies (USA); experiments were performed in compliance with the Argentinian animal protection laws and approved by the “Prof. Ricardo A. Margni” Humoral Immunity Studies Institute (IDEHU), National Research Council (CONICET-UBA).

### IA-2_ic_ expression as a fusion protein with thioredoxin in *E. coli*

#### Expression vector and *E. coli* transformation

**Fig. 1:**
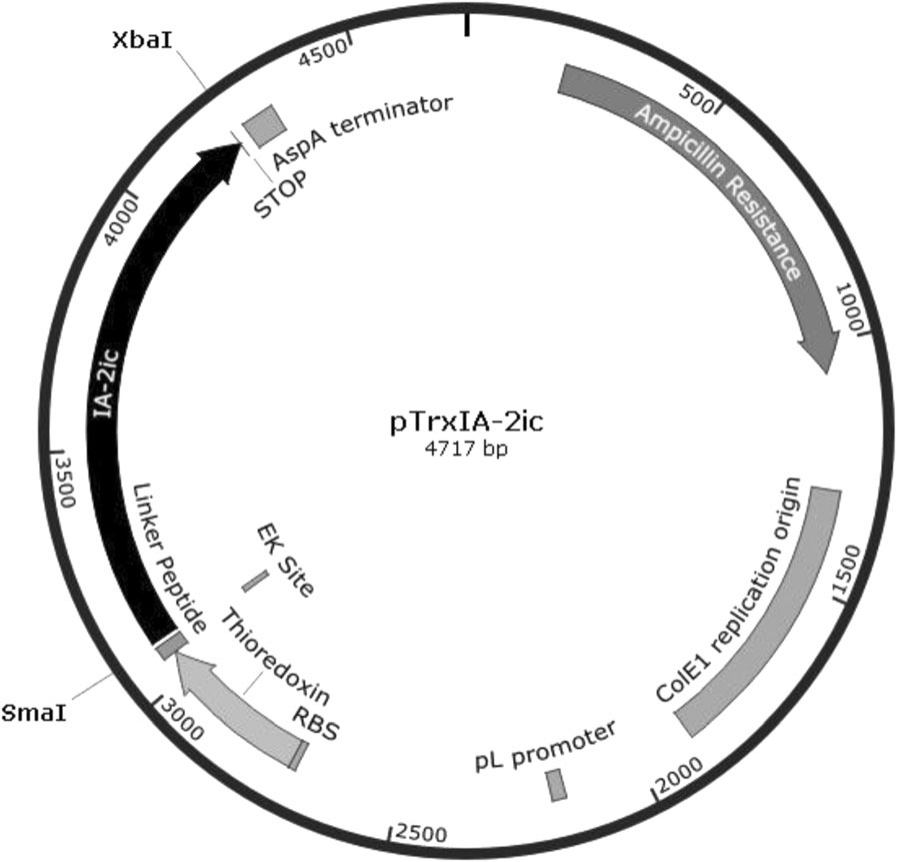
Map of the vector constructed for the expression of TrxIA-2_ic_ in *E. coli*. The pTrxFus vector was used to create a C-terminal fusion to *E. coli* thioredoxin. The IA-2_ic_ optimised sequence was inserted into the multiple cloning site of the expression vector and expressed as amino terminal fusion to the *E. coli* protein thioredoxin. This vector includes an enterokinase (EK) cleavage site that allows release of the native protein from Trx. To drive expression of thioredoxin fusions, pTrxFus uses the pL promoter from the λ bacteriophage and the AspA transcription terminator. Plasmid selection and maintenance is ensured by the presence of a beta-lactamase gene (*BLA*) that provides ampicillin resistance. *SmaI* and *XbaI* sites are indicated at the 3’ and 5’ ends of the IA-2_ic_ sequence. RBS: ribosome binding site

Unless otherwise indicated, standard molecular-biology protocols were used [52]. The coding sequence of human IA-2_ic_ (residues 604 to 979) was adapted from the sequences found in NCBI GenBank (L18983.1: Homo sapiens tyrosine phosphatase (IA-2/PTP) mRNA, complete cds). Human IA-2_ic_ codons were customized to those used with the highest frequencies in *E. coli* (codon optimization). The IA-2_ic_ optimized nucleotide sequence was synthesized by Genscript (GenScript Corporation, Piscataway, NJ, USA; www.GenScript.com), including *SmaI* and *XbaI* restriction sites at the 3’ and 5’ ends, respectively. The synthesized construct (1128 bp) was obtained from Genscript in plasmid pUC57 and maintained in JM109 *E. coli* (Promega, Madison, WI, USA). After vector propagation and purification with QIAprep spin Miniprep Kit (QIAGEN, Hilden, Germany), the IA-2_ic_ construct was released with *SmaI* and *XbaI* and ligated to the pTrxFus linearized vector (Invitrogen, Carlsbad, CA, USA) to yield pTrxIA-2_ic_ (Fig. 1). The quality of the new vector encoding the fusion protein TrxIA-2_ic_ was corroborated by sequencing (Macrogen Inc, Seoul, Korea). Competent *E. coli* GI698 (Invitrogen, Carlsbad, CA, USA) and GI724 (ATCC® 55151™) strains were transformed with pTrxIA-2_ic_ by electroporation.

#### Protein expression and *E. coli* disruption

Bacteria were cultured at 30 °C in 0.2 % w/v casein amino acids, 0.5 % w/v glucose, 1 mM MgCl_2_, and 100 μg/mL ampicillin, and protein expression was induced with 100 μg/mL tryptophan at 20 °C for GI698 or 37 °C for GI724. Different timepoints during the course of the induction period were collected; including 1.5, 3.0 and 16.0 h. Before and after the induction of protein expression, bacterial pellets from 1 mL culture were collected by centrifugation, suspended in 0.2 mL of SDS-PAGE sample buffer (50 mM Tris-HCI, 12 % w/v glycerol, 0.005 % w/v bromophenol blue, 4 % w/v SDS, 4 % v/v 2-mercaptoethanol -2ME-, pH 6.8) and boiled for 5 min to obtain the total cell lysate (TCL). A bacterial pellet was collected from 200 mL culture by centrifugation, suspended in 2 mL lysis buffer (50 mM Tris-HCl, 100 mM NaCl, 1 mM EDTA, pH 7.0) and sonicated in the presence of 1 mM 2ME and protease inhibitors (0.1 % w/v aprotinin and 2 mM phenylmethylsulfonyl fluoride) over crushed ice. After sonication, Triton X-100 was added to a final concentration of 0.1 % v/v and incubated for 10 min at 0 °C. The intracellular soluble fraction (ISF) was then separated by centrifugation at 15,000 rpm for 10 min at 4 °C.

### Purification by affinity chromatography

TrxIA-2_ic_ was purified by means of affinity chromatography following the protocol previously described [53]. The resin was based on an agarose support covalently modified with phenylarsine oxide (Additional file 1: Figure S1), which permitted the binding of proteins containing vicinal dithiol residues, as in Trx [54]. This resin, previously equilibrated in lysis buffer (≈4 mL) and activated with four column volumes (CV) of lysis buffer containing 20 mM 2ME, was added to the lysate (4 mL), and the resulting suspension was incubated for 1.5 h at 4 °C. The resin was poured into a column (0.7 cm × 9.0 cm) and washed sequentially with six CV lysis buffer, six CV lysis buffer containing 1 mM 2ME and three CV lysis buffer containing 5 mM 2ME. Bound proteins were eluted with several 2 mL of the lysis buffer containing 100 mM 2ME. The protein concentration in purified fractions was determined using the Coomasie Plus (Bradford) Assay kit (ThermoScientific, Rockford, IL, USA). Aprotinin was added to a final concentration of 0.1 % w/v.

### Biotinylation of TrxIA-2_ic_

Two mL of the purified fusion protein were subjected to buffer exchange to PBS using a ZEBA desalt spin column (Pierce Biotechnology, Rockford, IL, USA) according to the manufacturer’s instructions. The desalted protein was then incubated for 2 h at 0 °C with a 20-fold molar excess of sulfo-NHS-biotin (Pierce Biotechnology, Rockford, IL, USA). Free biotin was removed on a new ZEBA desalt spin column. Higher biotinylation rates were also tested with a 435-fold excess of sulfo-NHS-biotin.

### Sodium dodecyl sulphate-polyacrylamide gel electrophoresis and western blot analysis

TCL and ISF were analyzed by sodium dodecyl sulphate-polyacrylamide gel electrophoresis (SDS-PAGE) under reducing conditions, followed by Coomasie Brilliant Blue R-250 staining [55], and western blot (WB) [56]. For comparison, all SDS-PAGE lanes in each gel contained proteins recovered from the same amount of cells. Protein bands were transferred to nitrocellulose membranes and unoccupied binding sites were blocked by incubating with 3 % w/v skim milk in Tris buffer saline (TBS; 0.05 M Tris-HCl, 0.15 M NaCl, pH 7.5) for 2 h at room temperature (RT). After 3 washing steps with 0.05 % v/v Tween 20 in TBS (TBS-T) membranes were incubated ON at RT with polyclonal sera to Trx or IA-2 diluted 1/100 in 3 % w/v skim milk, 0.05 % v/v Tween 20 in TBS (TBS-MT), and then washed five times with TBS-T. Bound antibodies were visualized by incubation with peroxidase-conjugated goat antibodies to rabbit IgG (Jackson ImmunoResearch Laboratories, Inc., West Grove, PA) diluted 1/2000 in TBS-MT, followed by the addition of alpha-chloronaphthol (Sigma-Aldrich, Inc., St Louis, MO) and 10 vol H_2_O_2_.

### Biochemical characterization of TrxIA-2_ic_

#### Molecular weight determination by SDS-PAGE

The general SDS-PAGE procedure consisted in separating several conventional protein standards with known molecular weight (MW) (Amersham Pharmacia Biotech, Little Chalfont Buckinghamshire, England) in parallel with a TrxIA-2_ic_ sample in a 10 % T, 6 % C acrylamide gel with 1.5 mm thickness and under reducing conditions [55]. The standard proteins were used to generate a curve correlating MW and migration in the gel (relative mobility or R_f_), from which the MW of the TrxIA-2_ic_ sample was estimated.

#### Analytical size-exclusion chromatography

Size-Exclusion chromatography (SEC) was performed on an FPLC ÄKTA system (GE Healthcare, Sweden) equipped with a Superdex 75 HR 10/30 column (Pharmacia Biotech, Uppsala, Sweden). The chromatographic process was performed at RT with PBS as mobile phase at a flow rate of 1.0 mL/min and the absorbance was monitored at 280 nm. The MW, hydrodynamic dimension and aggregation state were assessed [57]. SEC calibration curves were generated, employing MW standard proteins (gel filtration standard, Bio-Rad, Hercules, CA, USA), using linear regression analysis of the plot of peak elution volume (V_e_) vs. the logarithm of their MW, obtained from chromatographic trace. In parallel, purified TrxIA-2_ic_ (50 μg/500 μL) was injected and the V_e_ obtained was interpolated in the calibration plot in order to estimate MW and aggregation state of the sample.

#### Mass spectrometry analysis

In order to assess total protein MW, affinity purified TrxIA-2_ic_was subjected to mass spectrometry (MS) analysis. Briefly, a 1 μL aliquot of the sample was spotted onto an AnchorChip (Bruker, Billerica, MA, USA). One μL of an oversaturated solution of sinapinic acid in 30/70/0.1 % v/v acetonitrile/water/TFA was added to the sample and left to crystallize by air-drying. Samples were analyzed on a Bruker Microflex MALDI-TOF device (Bruker, Billerica, MA, USA). In order to further characterize and identify the protein, TrxIA-2_ic_ was subjected to proteolytic digestion and MS analysis. Concisely, the sample was dissolved in 50 mM NH_4_HCO_3_ buffer, pH 8; a volume equivalent to 20 μg was subjected to disulfide bond reduction, with 20 mM dithiothreitol for 45 min at 56 °C, and alkylation with 20 mM iodoacetamide for 45 min at RT in the dark. The sample was then diluted to a final concentration of 1 M urea. Finally, trypsin and chymotrypsin proteolytic digestion were performed separately and samples were analyzed by nanoHPLC (EASY-Spray Accucore, Thermo scientific, West Palm Beach, FL, USA) coupled to a mass spectrometer with Orbitrap technology (Q-Exactive, Thermo Scientific, West Palm Beach, FL, USA; at Centro de Estudios Químicos y Biológicos por Espectrometría de Masa -CEQUIBIEM- CONICET-UBA, Argentina), enabling both separation and identification of peptides. Ionization of samples was made by electrospray (EASY-SPRAY, Thermo Scientific, West Palm Beach, FL, USA) and data analysis was performed by the Proteome Discoverer software version 1.4, Thermo Scientific. Coverage percentages were calculated based on the number of identified peptides/total number of peptides.

### Immunochemical characterization of TrxIA-2_ic_

#### Synthesis of [^35^S]IA-2 tracer

The [^35^S]IA-2 tracer was obtained by in vitro transcription/translation of the _c_DNA coding for human IA-2_ic_cloned into pSP64 vector (Promega, Madison, WI, USA), using a rabbit reticulocyte lysate system (Promega, Madison, WI, USA) in the presence of [^35^S]-methionine (New England, Nuclear, Boston, MA, USA), according to the manufacturer’s instructions. Translation products were diluted in radioimmunoassay (RIA) buffer (0.02 M Tris-HCl, 0.15 M NaCl, 0.1 % v/v Tween 20, pH 7.4) and applied to a PD10 column (Pharmacia-LKB Biotechnology, Uppsala, Sweden) in order to remove free [^35^S]-methionine. Typically, the percentage of incorporation of [^35^S]-methionine to the protein by this method was 5–7 %, yielding about 5–7×10^6^cpm of labelled protein. The tracer was stored in aliquots at -40 °C, and had a shelf life of 5 weeks.

#### Radioimmunoassay protocol

Quantitative competition assays were performed by standard Radioimmunoassay (RIA) protocols. The incubation of 2.5 μL of IA-2A(+) Type 1 diabetic patient sera with 10,000 cpm of [^35^S]IA-2 in the presence of serial concentrations (90.0 pM – 0.6 μM in a final volume of 60 μL) of purified TrxIA-2_ic_ from ISF was performed in duplicate. After ON incubation, 50 μL of 40 % v/v protein A-Sepharose 4B FF (GE Healthcare Biosciences, Uppsala, Sweden) in RIA buffer were added and incubated for 2 h at RT on an end-over-end shaker. Subsequently, samples were allowed to settle and the supernatants were discarded in order to isolate immunocomplexes. Pellets were washed three times with 200 μL of RIA buffer and once with 200 μL of 0.2 M NaCl in RIA buffer. Finally, pellets were suspended in 100 μL of 1 % w/v SDS and supernatants were carefully transferred to vials for scintillation counting (1 min/tube). Results for each sample were calculated as Bound% (B%) = 100 × (bound cpm/total cpm). Inhibitory Dose-response curves [log (inhibitor) vs. response - Variable slope (four parameters)] were fitted to the mathematical function:

$$ \mathrm{B}/{\mathrm{B}}_0=\mathrm{B}/{\mathrm{B}}_{0 \min }+\left(\mathrm{B}/{\mathrm{B}}_{0 \max }{\textstyle \hbox{-}}\mathrm{B}/{\mathrm{B}}_{0 \min}\right)/\left(1+1{0}^{\left[\left( \log \kern0.5em \mathrm{I}{\mathrm{C}}_{50}{\textstyle \hbox{-} } \log \kern0.5em \mathrm{TrxIA}{\textstyle \hbox{-} }{2}_{\mathrm{ic}}\kern0.5em \mathrm{dose}\right)\ast \mathrm{Hill}\kern0.5em \mathrm{Slope}\right]}\right) $$

Where B corresponds to B% measurements, B_0_ is the B% at zero concentration of unlabelled antigen, B/B_0min_ and B/B_0max_ are the minimal and maximal response, respectively and the parameter IC_50_ represents the concentration of TrxIA-2_ic_ that gave a response half between B/B_0min_ and B/B_0max_. Hill Slope describes the steepness of the family of curves. The same protocol was performed for TrxIA-2_ic_-biotin, in serial concentrations of 12.0 pM – 0.7 μM.

#### Inhibition assay

The ability of TrxIA-2_ic_ to compete with [^35^S]IA-2 for the binding to antibodies was assessed qualitatively by incubating sera from 30 IA-2A(+) Type 1 diabetic patients with the tracer in the presence of TrxIA-2_ic_ excess (190 nM). Briefly, 2.5 μL of human sera were incubated ON at 4 °C with 10,000 cpm of [^35^S]IA-2 in the absence or presence of unlabelled TrxIA-2_ic_ in a final volume of 60 μL in RIA buffer. Immunocomplexes were isolated with protein A-Sepharose 4B FF; pellets were washed and suspended in 1 % w/v SDS, as described in the RIA protocol. The radioactivity of supernatants was counted. Results for each sample (with or without TrxIA-2_ic_) were calculated as B%, and expressed as Standard Deviation scores (SDs) = (B% - B_c_%) /SD_c_, where B_c_% is the mean B% of healthy control sera and SD_c_ its standard deviation. The same protocol was followed for TrxIA-2_ic_-biotin in the presence of antigen excess (140 nM).

### Storage and stability studies

Inhibition dose-response curves were performed over time as previously described. Briefly, a 2.5 μL aliquot of a pool of sera from 5 IA-2A (+) Type 1 diabetic patients was incubated with the tracer [^35^S]IA-2 and several dilutions of purified TrxIA-2_ic_ in two different storage conditions. These conditions included TrxIA-2_ic_ stored at -20 °C with or without the addition of glycerol to a final concentration of 50 % v/v in lysis buffer either with 100 mM 2ME or 50 mM 2ME, respectively. The study was performed over the course of 120 days and dose-response curves of log [TrxIA-2_ic_] vs. B/B_0_ were analyzed at each time for both conditions. The parameter IC_50_ was selected for comparison and selection of the optimal storage condition.

### TrxIA-2_ic_ application in immunoassays for IA-2A assessment

#### Reagents

PBS was used as the microplate coating buffer. Two percent w/v skim milk in PBS (PBS-M) and PBS containing 0.05 % v/v Tween 20 (PBS-T) were used as blocking solution and washing buffer, respectively. Sample or reagent dilutions were prepared in 2 % w/v skim milk, 0.05 % v/v Tween 20 in PBS (PBS-MT). Immunopure Avidin was purchased from Pierce, and Avidin–Horseradish Peroxidase (HRP) and rabbit anti-human IgG-HRP were purchased from Jackson ImmunoResearch Laboratories, Inc. The 3,3’,5,5’-tetramethyl-benzidine/H_2_O_2_ (Single Component TMB Peroxidase EIA Substrate Kit, BioRad, Hercules, CA, USA) was employed as the chromogenic substrate and the SuperSignal ELISA Pico (ThermoScientific, Rockford, IL, USA) as the chemiluminescent substrate. Except when otherwise indicated, incubations were performed at RT, washing steps were performed with PBS-T and 50 μL per well were added in each incubation step.

#### Radioligand binding assay (RBA) protocol

RBA was performed as previously described [43] with minor modifications [50, 58]. Briefly, 2.5 μL of human sera were incubated ON at 4 °C with 10,000 cpm of [^35^S]IA-2 in a final volume of 60 μL in RIA buffer. After that, 50 μL of 40 % v/v protein A-Sepharose 4B FF in RIA buffer were added and incubated for 2 h at RT on an end-over-end shaker. Subsequently, samples were allowed to settle and the supernatants were discarded in order to isolate immunocomplexes. Pellets were washed three times with 200 μL of RIA buffer and once with 200 μL of 0.2 M NaCl in RIA buffer. Finally, pellets were suspended in 100 μL of 1 % w/v SDS and supernatants were carefully transferred to vials for scintillation counting (1 min/tube). Results for each sample were calculated as Bound% (B%) = 100 × (bound cpm/total cpm), and expressed as Standard Deviation scores (SDs) = (B% - B_c_%) /SD_c_, where B_c_% is the mean B% of healthy control sera and SD_c_ its standard deviation. Samples were considered positive when SDs > 3. This assay had 66.0 % sensitivity, 97.8 % specificity and 86.43 % accuracy in the Islet Autoantibody Standardization Program (IASP) 2015, laboratory 0519.

#### Blank corrected ELISA with colorimetric detection (_bc_ELISA)

The protocol employed was based on those previously described [50, 59–61], with minor modifications. Briefly, polystyrene microplates (Maxisorp, NUNC, Roskilde, Denmark) were coated ON at 4 °C with 0.50 μg purified avidin per well, in coating buffer and washed five times with PBS. Blocking solution (200 μL/well) was added, and plates were incubated for 1.5 h. Microplates were washed three times, and incubated for 4 h at 4 °C with 20.0 ng of TrxIA-2_ic_-biotin. After washing six times, duplicate serum samples diluted 1/10 were added and incubated ON at 4 °C. To measure the non-specific signal, duplicates of serum samples were incubated in wells without TrxIA-2_ic_-biotin. Plates were washed six times, and bound antibodies were detected with anti-human IgG-HRP (diluted 1/20,000, 1.5 h at 4 °C). After washing (five times plus one final wash with PBS), the chromogenic substrate was added and plates were incubated in the dark. The color reaction was stopped with 2 M H_2_SO_4_. The oxidized substrate was measured at 450 nm with an ELISA plate reader Multiskan EX (Thermo electron corporation, Vantaa, Finland). Results were calculated as specific absorbance (A_s_, mean of each sample minus the mean of their non-specific control), and expressed as SDs = (A_s_-A_c_)/SD_c_, where A_c_ is the mean specific absorbance of healthy control sera and SD_c_ its standard deviation. The cut-off value of the assay was set at SDs = 1.6.

#### Bridge ELISA with chemiluminescent detection (CL-_b_ELISA)

The protocol employed was based on those previously described [62–65]. White Polystyrene microplates (96 F Maxisorp white microwell, Thermoscientific, Rockford, IL, USA) were coated ON at 4 °C with 40.0 ng of purified TrxIA-2_ic_ per well, washed three times with PBS, blocked for 1.5 h with 200 μL of blocking solution, and washed five times. Samples were added in duplicate and microplates were incubated for 1 h. Plates were then washed five times and 2.0 ng of TrxIA-2_ic_ -biotin per well were added (the TrxIA-2_ic_-biotin employed was synthesized with high rates of biotinylation). After another 1 h incubation, plates were washed five times and bound TrxIA-2_ic_ -biotin was detected by the addition of Avidin-HRP diluted 1/500. After 1 h incubation, microplates were washed four times plus one final washing step with 200 μL of PBS; the chemiluminescent substrate was added and incubated 1 min in the dark. The chemiluminescent reaction was measured in a Victor^3^ multilabel reader (Perkin Elmer, MA, USA). Results were calculated as specific counts per second (cps_s_, mean of each sample minus the mean of blank wells), and expressed as SDs = (cps_s_ - cps_c_) /SD_c_, where cps_c_ is the mean specific cps of healthy control sera and SD_c_ its standard deviation. Samples were considered positive when SDs > 2.5.

### Statistical analysis

The identity and parallelism between inhibitory dose-response curves obtained with TrxIA-2_ic_ and TrxIA-2_ic_-biotin were analyzed by comparing B/B_0min_, B/B_0max_, Hill slopes and IC_50_ values by the extra sum-of-squares F test comparison method. Normal distribution of data was analyzed by the D’Agostino and Pearson omnibus normality test. In order to remove outliers from normally distributed healthy control individuals, the Rout test was performed. The selection of optimal cut-off values was based on curves constructed by plotting the calculated specificity and sensitivity of each protocol vs. the corresponding cut-off values. Statistical significance was evaluated using parametric tests: paired samples Student *t* test and unpaired samples Student *t* test with Welch correction, or non-parametric tests: Wilcoxon matched-pairs signed rank test or Mann-Whitney *U*-test for unpaired data, when applicable. The correlation between methods for IA-2A assessment was calculated using the non-parametric Spearman Rank Correlation. Calculations were performed using GraphPad Prism version 6.01 for Windows (GraphPad Software, San Diego California, USA, www.graphpad.com). A *p*-value < 0.05 was considered statistically significant.

## Results

### IA-2_ic_ expression as a fusion protein with thioredoxin in *E. coli*

**Fig. 2:**
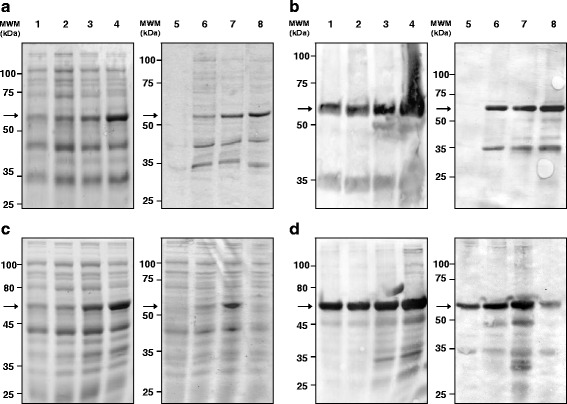
IA-2_ic_ expression as a fusion protein with Trx in *E. coli* GI698 and GI724. **a** and **c**: SDS-PAGE (12.1 % T 6.0 % C, 1 mm, under reducing conditions, stained with Coomassie Brilliant Blue R-250), **b** and **d**: WB revealed with a rabbit polyclonal serum to thioredoxin as primary antibody. Samples: Total Cell Lysate for **a** and **b**, Intracellular Soluble Fraction for **c** and **d**. *Lanes 1-4*: samples from pTrxIA-2_ic_ transformed *E. coli* GI698 strain; *lanes 5-8*: samples from pTrxIA-2_ic_ transformed *E. coli* GI724 strain. *Lanes 1* and *5*: sample before induction (0 h); *lanes 2* and *6*: sample after 1.5 h of induction; *lanes 3* and *7*: sample after 3.0 h of induction; *lanes 4* and *8*: sample after 16.0 h of induction. *Arrows* indicate the electrophoretic mobility of TrxIA-2_ic_

Competent *E. coli* GI698 and GI724 strains were transformed with pTrxIA-2_ic_ by electroporation. Protein expression was induced with Trp in both strains at different incubation times and temperatures (20 °C and 37 °C for GI698 and GI724, respectively) in order to achieve optimal conditions for maximal protein production. For this analysis, SDS-PAGE and WB for TCL were carried out with specific protein recognition by polyclonal serum to Trx. Figure 2a and b depicts bands compatible with TrxIA-2_ic_ theoretical MW (≈55.4 kDa) in both bacteria strains, whereas such bands are absent in TCL from non-transformed bacteria under the same experimental conditions. Maximal protein expression was detected at 16 h post-induction in TCL from both strains. In order to select the optimal temperature and time for induction, bacteria were harvested and lysed as previously described. The ISF was isolated from both strains at each induction time, and analyzed by SDS-PAGE and WB (Fig. 2c and d). Relative protein expression was compared at 3.0 h after induction, where GI724 strain showed slightly higher values than GI698 as regards the ≈ 55.4 kDa band from the total protein mass. Furthermore, almost total loss of protein from ISF in GI724 strain was observed at 16.0 h after induction.

### Purification of TrxIA-2_ic_

**Fig. 3:**
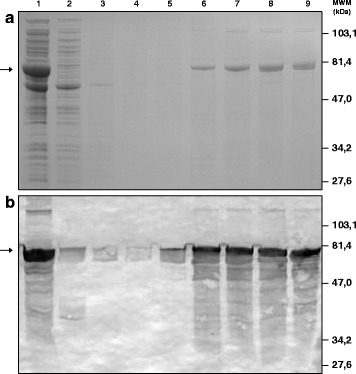
Purification of TrxIA-2_ic_by affinity chromatography. Analysis of TrxIA-2_ic_ fractions at different stages of purification by **a**: SDS-PAGE (10.0 % T 6.0 % C, 1 mm thickness, under reducing conditions, stained with Coomassie Brilliant Blue R-250) and **b**: WB revealed with a rabbit polyclonal serum to thioredoxin as primary antibody. In A and B, lane 1 corresponds to total ISF from transformed *E. coli* GI724, lane 2: unbound material or pass through of the column, lanes 3-5: washes with increasing 2ME concentrations (0, 1 and 5 mM), lanes 6-9: 100 mM consecutive eluates of purified TrxIA-2_ic_. Arrows indicate the electrophoretic mobility of TrxIA-2_ic_

TrxIA-2_ic_ was purified from the ISF of *E. coli* GI724 lysate after 3 h of induction. The procedure was based on affinity chromatography in which the Trx portion of TrxIA-2_ic_ binds to the resin by its catalytic domain containing vicinal dithiols. Figure 3 depicts SDS-PAGE and WB of different stages of purification, with high levels of protein expression revealed (lane 1). One-step purification separated most contaminant proteins with little or no significant loss of TrxIA-2_ic_ relying on the high capacity of the in-house made resin. Based on the quantification of the 100 mM 2ME fraction (lanes 6–9) bands, the purification yielded ≈ 10 mg of 72–77 % pure TrxIA-2_ic_/L of culture medium.

### Biochemical characterization of TrxIA-2_ic_

**Fig. 4:**
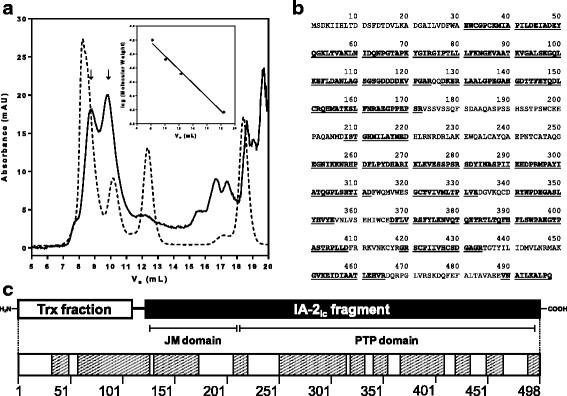
Biochemical characterization of TrxIA-2_ic_. **a**: Size Exclusion Chromatography of affinity-purified TrxIA-2_ic_ (*solid line*) and molecular weight globular calibrators (*dotted line*) performed on a Superdex 75 column (insert: calibration curve). The detector was set at 280 nm. From left to right, calibrator peaks correspond to human gamma-globulin (158 kDa), ovalbumin (44 kDa), horse myoglobin (17 kDa) and B_12_ vitamin (1.35 kDa). *Arrows* indicated peaks from monomeric (V_e_ = 9.793 mL) and dimeric TrxIA-2_ic_ (V_e_ = 8.753 mL). **b**: TrxIA-2_ic_ sequence. *Underlined* and *highlighted* in *bold* are those fragments identified by Orbitrap Mass Spectrometry considering high confidence peptides. **c**: TrxIA-2_ic_ N-terminal Trx fraction, the linker peptide and the IA-2_ic_ fragment in the C-terminal of the construction are detailed in the scheme above, and the corresponding peptide fragments identified by Orbitrap Mass Spectrometry are highlighted in the diagram below. JM: Juxtamembrane

In order to estimate the MW of TrxIA-2_ic_, an SDS-PAGE with MW calibrators (phosforilase b, bovine albumin, ovalbumin, carbonic anhydrase, trypsin inhibitor and alpha lactalbumin) was performed. A calibration curve of log MW vs. R_f_ was constructed (log MW = -0.9091 × R_f_ + 4.9932, R^2^ = 0.9932) and the unknown MW of TrxIA-2_ic_ was interpolated. An experimental MW (55,943 Da) compatible to the theoretical MW (55,358 Da) was obtained (1.1 % error, which is a satisfactory accuracy for this method) [66]. Moreover, further MW studies were carried with SEC in order to characterize TrxIA-2_ic_ hydrodynamic behavior and the presence of aggregates (Fig. 4a). These results revealed the presence of monomeric and dimeric forms of TrxIA-2_ic_ with the corresponding estimated MW (61,187 Da and 98,047 Da, respectively). Values were obtained from a calibration curve of log MW vs. V_e_ (log MW = -0.1969 × V_e_ + 6.7149, R^2^ = 0.9906). For TrxIA-2_ic_ identification in eluted fractions, a WB using rabbit polyclonal serum to IA-2 was performed, confirming the presence of the fusion protein at expected monomeric and dimeric MW. In fact, by subjecting the dimer to reduction with dithiothreitol, total conversion into the monomer state was observed (Additional file 2: Figure S2). Moreover, SEC showed a TrxIA-2_ic_ Stokes radius of 33.2 Å for the monomer peak, which corresponded to a globular protein of 64,816 Da.

MS analysis of TrxIA-2_ic_ corroborated the agreement between the chimera MW and the expected value (actual mass 55,209.008 Da; theoretical mass 55,357.500 Da) (Additional file 3: Figure S3). The purified fusion protein was also subjected to proteolytic digestion with trypsin and chymotrypsin. The peptide profile obtained after digestion was analyzed by Orbitrap mass spectrometry, achieving 63.25 % total coverage (38.15 % for trypsin and 42.77 % for chymotrypsin) of TrxIA-2_ic_ (498 residues) (Fig. 4b and c), considering high confidence peptides only; when low confidence peptides were not filtered, 95.38 % coverage of the theoretical composition of the protein was accounted for.

### Immunochemical characterization of TrxIA-2_ic_

**Fig. 5:**
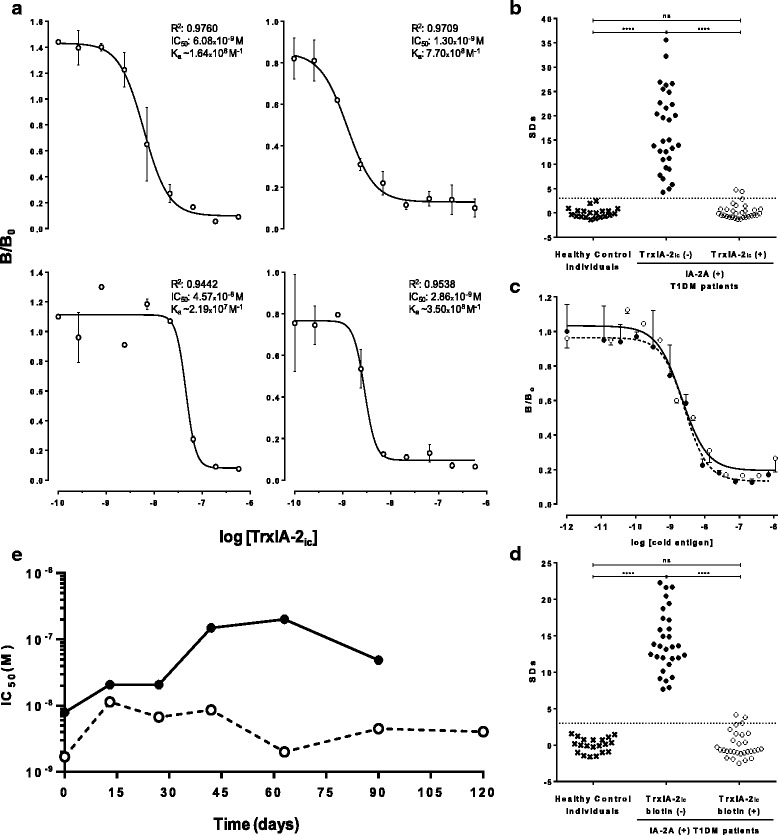
Immunochemical characterization and storage study. **a**: Dose-response curves for 4 IA-2A RBA positive Type 1 diabetic patient sera incubated with different concentrations of TrxIA-2_ic_. Each curve includes best-fit values from the log (inhibitor) vs. response - Variable slope (four parameters) equation: correlation coefficient data (R^2^), IC_50_ values and an approximation to the affinity constant of IA-2A (K_a_) calculated as the inverse of IC_50_. **b**: Inhibition capacity of TrxIA-2_ic_ assessed in 30 IA-2A positive type 1 diabetic patient sera in the absence (*left*) or presence (right) of TrxIA-2_ic_. Twenty healthy control sera were used in order to set a cut-off value. **c**: Dose-response curves for a pool of 3 IA-2A RBA positive Type 1 diabetic patient sera for different concentrations of TrxIA-2_ic_ (*open circle*, *solid line*) and TrxIA-2_ic_-biotin (*closed circle*, *dotted line*). **d**: Inhibition capacity of TrxIA-2_ic_-biotin assessed in 30 IA-2A positive Type 1 diabetic patient sera in the absence (*left*) or presence (right) of TrxIA-2_ic_-biotin. Twenty healthy control sera were used in order to set a cut-off value. Binding is expressed as SDs, and the dotted lines represent the cut-off value. **e**: IC_50_ vs. storage time for TrxIA-2_ic_ stored at -20 °C in 100 mM 2ME lysis buffer (*solid line*) and -20 °C in 50 % glycerol, 50 mM 2ME lysis buffer (*dotted line*). These assays were performed by standard RIA protocols, as described in Methods.

Dose-response curves with 4 IA-2A RBA positive Type 1 diabetic patient sera were performed by RIA, adding TrxIA-2_ic_ at variable concentrations and using [^35^S]IA-2 synthesized in an eukaryotic system (Fig. 5a). All dose-response curves (log [TrxIA-2_ic_] vs. B/B_0_) showed similar IC_50_ (ranging from 4.57×10^-9^ to 1.30×10^-8^ M), indicating comparable TrxIA-2_ic_ immunoreactivity with sera. The immunochemical ability of TrxIA-2_ic_ to compete with [^35^S]IA-2 was assessed qualitatively by incubating 30 Type 1 diabetic patients sera in the presence of 190 nM TrxIA-2_ic_ (Fig. 5b). All tested sera were IA-2A positive with SDs of 17.00 ± 8.29 (mean ± SD), median 14.90, range 4.24 to 35.09 and cut-off value for positivity SDs = 3.0. A significant difference between sera from Type 1 diabetic patients and healthy control individuals was observed (Unpaired samples Student *t* test with Welch correction, *p* <0.0001). All type 1 diabetic patient sera became virtually negative under antigen excess (comparable to healthy control individuals SDs): median SDs changed from 14.90 to -0.16 (range -1.31 to 4.73) with cold TrxIA-2_ic_ excess (Wilcoxon test for paired samples, *p* <0.0001).

Competitive quantitative and qualitative assays were also performed for TrxIA-2_ic_-biotin in order to evaluate any distortion in epitopes caused by biotynilation. The immunochemical identities between TrxIA-2_ic_ and TrxIA-2_ic_-biotin were analyzed and expressed in terms of parallelism. As seen in Fig. 5c, when using a pool of IA-2A+ human sera, parallelism and identity between curves was achieved (one curve adequately fits all data, alpha = 0.05, *R* ^2^ = 0.9492). Similar results were obtained for TrxIA-2_ic_ biotinylated under conditions of high biotin concentrations (Additional file 4: Figure S4). The inhibition assay for TrxIA-2_ic_-biotin was performed by incubating 30 Type 1 diabetic patient sera in the presence of 140 nM TrxIA-2_ic_-biotin (Fig. 5d). All tested sera were IA-2A positive with an SDs of 14.16 ± 4.17 (mean ± SD), median 13.53, range 7.68 to 22.27 and a cut-off value for positivity SDs = 3.0. It can be observed that sera from Type 1 diabetic patients differed significantly from healthy control samples (Unpaired *t* test with Welch correction, *p* <0.0001). All patient sera became virtually negative (comparable to healthy control individuals) under antigen excess: mean SDs changed from 14.16 to 0.01 (median -0.67, range -2.52 to 4.13) with cold TrxIA-2_ic_-biotin excess (Student *t* test for paired samples, *p* <0.0001).

### Storage and stability studies

Displacement curves were performed over time (120 d) using a pool of 5 sera from IA-2A positive Type 1 diabetic patients and the tracer [^35^S]IA-2 in competition with cold TrxIA-2_ic_ under different storage conditions. Inhibition dose response curves (log [TrxIA-2_ic_] vs. B/B_0_) were drawn for each condition and the IC_50_ value was determined and plotted against time (Fig. 5e). As it can be observed, the IC_50_ values remained relatively constant over time for the storage condition of -20 °C in 50 % v/v glycerol, 50 mM 2ME lysis buffer, indicating that the immunoreactivity was maintained throughout the evaluation period. Contrarily, the storage condition at -20 °C in 100 mM 2ME lysis buffer caused a gradual increase in the IC_50_ value until *t* = 120 d, when the dose-response curve could no longer be properly fitted.

### TrxIA-2_ic_ application in immunoassays for IA-2A assessment

**Fig. 6:**
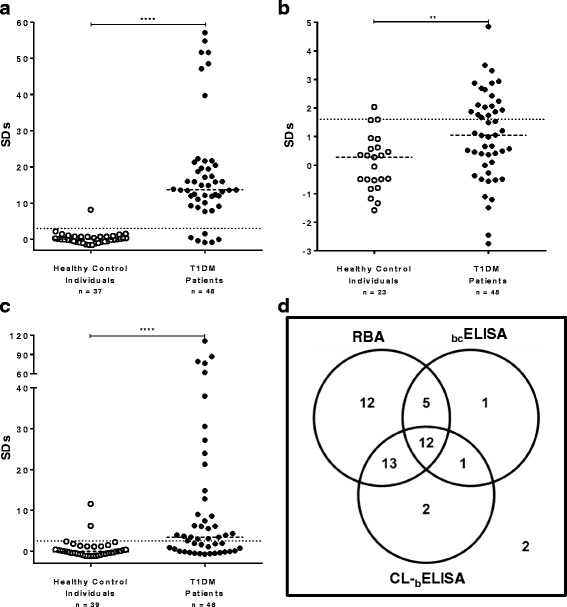
Immunoassays for IA-2A assessment in healthy control and Type 1 diabetic patient sera. The results, expressed as SDs, were obtained by RBA (**a**), _bc_ELISA (**b**) and CL-_b_ELISA (**c**). The cut-off value for each assay is indicated by a dotted line and medians for each population are indicated by a dashed line. **d**: Venn’s diagrams for integrated IA-2A results for children and adolescents with newly diagnosed T1DM, obtained by RBA, _bc_ELISA, and CL-_b_ELISA

Forty eight sera from children and adolescents with T1DM were tested in parallel by RBA, _bc_ELISA and CL-_b_ELISA for IA-2A detection. The specificity was calculated as 100 % minus the percentage of false positives, using control individuals (*n* = 37, 23 or 39 for RBA, _bc_ELISA and CL-_b_ELISA, respectively). In order to establish normally distributed results for healthy control individuals, outlier removal was needed (Rout test, *Q* = 1 %); however, these outliers were included in all plots and also for specificity calculations, as they were considered false positives. When analyzed by RBA, 42 sera were positive (87.5 %), while 6 were negative (12.5 %). A median SDs of 13.74 was obtained, ranging from -0.84 to 57.08; cut-off value for positivity SDs = 3.0 (Fig. 6a and Table 1). With this analysis, a specificity of 97.3 % was obtained and healthy control individuals differed significantly from T1DM patients (*p* < 0.0001).

**Table 1:** Analytical parameters from RBA, _bc_ELISA and CL-_b_ELISA

Assay	RBA	_bc_ELISA	CL-_b_ELISA
Healthy control individuals (*n*)	37	23	39
Type 1 diabetic patients (*n*)	48		
Median (SDs)	13.74	1.05	3.46
Range (SDs)	(-0.84)-57.08	(-2.76)-4.85	(-0.71)-111.20
Sensitivity^a^ (%)	87.5	39.6	58.3
Specificity^b^	97.9	95.7	94.9
Analytical Sensitivity^c^ (%)	-	40.5	59.5

^a^Percentage of IA-2A positive patients from the total population studied

^b^100 % minus the percentage of false positives

^c^Percentage of patients RBA positive that were positive by each method

Results were obtained from healthy control and newly diagnosed T1DM patient sera (*n* = 48).

By _bc_ELISA, a total of 19 sera out of the 48 T1DM patients analyzed were positive (39.6 % sensitivity), with SDs ranging from -2.76 to 4.85, and an SDs = 1.01 ± 1.55 (mean ± SD), median 1.05 and cut-off value for positivity SDs = 1.6. Out of the 42 IA-2A positive sera by RBA, 17 scored positive by _bc_ELISA, whereas 29 patients were negative (Fig. 6b). These results indicated that _bc_ELISA had an analytical sensitivity of 40.5 % for this sera collection (percentage of patients RBA positive that were positive by _bc_ELISA) and 95.7 % specificity when analysis of healthy control sera was performed (Table 1). It is noteworthy that the RBA failed to render positive reactions in 2 patients that were indeed detected by the _bc_ELISA. Populations significantly differed for median antibody levels (*p* = 0.0032).

Out of the 48 patient sera, 28 scored positive for IA-2A by CL-_b_ELISA (58.3 % sensitivity), whereas 20 patients were negative (41.7 %) (Fig. 6c). Twenty five IA-2A positive sera by RBA scored positive when evaluated by CL-_b_ELISA, showing an analytical sensitivity of 59.5 %, SDs ranging from -0.71 to 111.20 and a median SDs = 3.46 (Table 1). Healthy control sera (*n* = 39) resulted in 94.9 % specificity and showed two different statistical populations that needed outliers removal by the Rout test (*Q* = 1 %). Remaining results were normally distributed. In order to achieve maximum analytical sensitivity for this protocol, SDs > 2.5 was chosen as the cut-off value. As seen for _bc_ELISA, CL-_b_ELISA detected 3 positive sera that the RBA could not, one of which was positive for _bc_ELISA as well. Populations significantly differed for median antibody levels (*p* < 0.0001).

The Venn’s diagram (Fig. 6d) depicts the integrated results of RBA, _bc_ELISA, and CL-_b_ELISA. As shown on this figure, 12 out of 48 sera were positive by RBA, _bc_ELISA, and CL-_b_ELISA, while 2 were negative by all methods. Five sera were only positive by both RBA and _bc_ELISA, while 13 were only positive by both RBA and CL-_b_ELISA. Moreover, _bc_ELISA and CL-_b_ELISA were able to detect 4 patients that were found negative by RBA (1 serum found positive only by _bc_ELISA, 2 sera positive only by CL-_b_ELISA and 1 sera positive by both methods). The latter sera were not considered false positives since these samples had been obtained from newly diagnosed patients with T1DM. CL-_b_ELISA was able to detect 15 IA-2A positive sera that _bc_ELISA was not; whereas _bc_ELISA detected 6 patients as IA-2A positive that CL-_b_ELISA did not. The Spearman correlation coefficient (*r* = 0.6650 for _bc_ELISA and CL-_b_ELISA) and p value (*p* = 0.0005) showed that the pairing was significantly effective and there was an acceptable concordance between both methods; however, no significant correlation between antibody levels by RBA and either solid phase tests was found.

## Discussion

Although radiometric methods have an excellent performance in the diagnosis of DM, the use of radioactive isotopes is falling out of use because of their multiple disadvantages (high costs, waste management difficulties, low applicability in laboratories, centralization of determinations, etc.). Therefore, the development of alternative methods of acceptable performance, greater simplicity, lower cost and more applicability in low complexity laboratories are needed. The methods most widely used include solid phase immunoassays which require large amounts of antigen for their development and routine application. Our laboratory has previously attempted to produce recombinant IA-2_ic_ in order to implement ELISA-like tests [50]; however, due to the instability of the product (Poskus E, personal communication) the RBA for IA-2A remains the method of choice for routine analysis in Argentina.

In this work, an alternative approach for IA-2_ic_ expression was developed. The methodology presented herein involved the production of soluble cytoplasmic IA-2_ic_ by means of the solubilising effect of Trx [49], which is a phenomenon already observed for other proteins [67–70]. The use of the Trx fusion partner increases the chance of correct folding, which is critical for binding of autoantibodies from patients with T1DM. The expression of TrxIA-2_ic_ was accomplished in *E. coli* GI698 and GI724 strains (Fig. 2). High production levels of the C-terminal fusions to thioredoxin were obtained through the use of pTrxFus vector carrying pL, which is a strong bacteriophage λ promoter. As advised, collection of samples at different timepoints during the course of the induction period was carried out in order to evaluate variations in protein solubility, stability and concentration [49, 71]. The lysis of harvested bacteria was performed and the ISF evaluation was carried out (Fig. 2). Even though a substantial expression of TrxIA-2_ic_ was observed in both strains, GI724 showed a slightly higher protein expression under shorter induction periods than GI698. The expression of soluble TrxIA-2_ic_ became minimal under ON incubations in the GI724 strain, which was expected for large induction times under higher temperatures. It can be speculated that under such conditions, TrxIA-2_ic_ became unstable due to the excessive synthesis and improper folding, a phenomenon that led to insolubilisation and product accumulation in the form of inclusion bodies (IB). This observation was corroborated by SDS-PAGE and WB of isolated IB (Additional file 5: Figure S5). On the other hand, the expression in GI698 strain rendered good levels of protein, with a maximum at 16 h of induction, and minimal visualization of degradation by-products, probably due to low activity of proteases at 20 °C. Although GI698 strain should not be completely ruled out as a valuable source of TrxIA-2_ic_, shorter induction times are preferred for scaling up procedures. Taking into account these results, the expression in GI724 strain employing a 3 h induction time, was selected as optimal condition for TrxIA-2_ic_ production in any low-medium complexity laboratory and was therefore used for further studies.

The purification of TrxIA-2_ic_ from the ISF was accomplished by a single step of affinity chromatography using an agarose-based matrix with arsenic residues. This chemical ligand binds selectively to vicinal dithiols, such as the ones present in the catalytic site of Trx (binding motif Cys-Gly-Pro-Cys). The levels of soluble protein expression obtained in *E.coli* GI724 were higher than those previously reported in the literature for prokaryotic systems [72, 73]. High purity levels were achieved, even higher than those reported in the results section (72–77 %), which does not include the contribution of TrxIA-2_ic_ dimer. Furthermore, these unique properties of the fusion partner allowed us to establish a general platform for expression and purification of soluble Trx fusion proteins, such as TrxGAD and TrxPI [68, 70], along with TrxIA-2_ic_, applicable to the laboratory diagnosis of autoimmune DM. The amount of protein obtained with this methodology was suitable for the development of solid phase IA-2A immunoassays; in fact, the result of a standard purification protocol may allow passive adsorption of more than 500 96-well polystyrene microplates. Taking into account these results, the affinity purified TrxIA-2_ic_ from ISF was further characterized.

The first quality control step was to verify if TrxIA-2_ic_ had the theoretical expected MW. The analysis of the purified protein by SDS-PAGE, SEC and MS rendered results that coincided, within the experimental error, with the theoretical MW values calculated from the sequence. Furthermore, we observed that under native conditions, both a monomeric form and aggregates of a dimer-like conformation would coexist when solubilised. The SDS-PAGE analysis employing dithiothreitol demonstrated that these dimers were bound by covalent bonds, specifically disulfide bonds (Additional file 2: Figure S2). According to literature [74], the cysteine residues were unlikely to form disulfide bonds, contributing little to the three dimensional structure of the protein. Probably, disulfide bridges are formed later due to the action of reactive oxygen species during storage or the IA-2_ic_ PTP and juxtamembrane domains tendency to dimerize [75–77], leading to polymerization. This in vitro observation could be a resemblance of the in vivo regulation of many receptor-type PTPs. Furthermore, the oxidation of the core cysteine (C909) to cysteic acid would block access of antibodies, which turns storage buffer a critical aspect in the stability of IA-2_ic_ [78]. For this reason, we decided to maintain the 2ME reducing conditions after purification. Regarding the determination of the Stokes radius of the protein, since TrxIA-2_ic_ is a 55,358 Da protein, the hydrodynamic behavior was compatible with a properly folded monomer that deviates moderately from an ideal sphere (different elution behaviour between the standard and the unknown proteins) [66]. Upon performing MS analysis after proteolytic digestion, sequence coverage of 63.25 % was achieved over the 498 residues constituting the TrxIA-2_ic_, which is a percentage suitable to accomplish the autoantigen identification. Taking into account the biochemical characterization of the entire protein, we have expressed genuine human IA-2_ic_ as a fusion protein with Trx.

Immunochemical studies of TrxIA-2_ic_ allowed us to demonstrate that the protein is able to displace the binding of radiolabeled eukaryotic IA-2_ic_ from IA-2A, thus confirming the integrity of conformational epitopes in the recombinant antigen, which is a critical requirement to IA-2A recognition. It is noteworthy that the approximate K_a_ values obtained for 4 sera were in the order of 10^8^ M^-1^, which correlates with literature data (10^7^–10^11^ M^-1^) [79]. Since the calculated K_a_ for IA-2A were, within the limits of experimental error, the same for TrxIA-2_ic_ and these previously published data for IA-2_ic_ from *E. coli*, it can be speculated that the epitopes as well as the overall protein conformation are not affected during prokaryotic expression and Trx fusion. Furthermore, with a larger number of IA-2A positive patient sera, we demonstrated that they were able to recognize recombinant TrxIA-2_ic_, displacing the radioactively labelled eukaryotic antigen. Considering that most sera from type 1 diabetic patients only target conformational discontinuous epitopes of IA-2_ic_ [29], this result reinforces the hypothesis of a correct folding of epitopes in the IA-2_ic_ portion, probably due to the presence of energetically or kinetically favourable conditions.

Even though an EK site was included in the plasmid, a further enzymatic removal of the Trx portion from the fusion protein to avoid cross reaction phenomena was not necessary since we have previously demonstrated that healthy control and diabetic sera do not react with bacterial Trx [53]. Therefore, we have decided to use the entire fusion protein for the development of immunoassays since Trx did not interfere with IA-2A recognition, it helped in proper protein folding and stability, and even simplified purification. Furthermore, the amino group of the N-terminal portion of Trx could be useful to improve the orientation of IA-2_ic_ on surfaces with free carboxyl residues by covalent bonds, such as those required in Surface Plasmon Resonance CM5 sensor chips for thermodynamic and kinetic studies of autoantibodies, and in polystyrene microspheres for flow cytometry [53, 80].

In order to increase the possibilities for immunoassay development, TrxIA-2_ic_ was biotinylated. When the immunochemical behavior of TrxIA-2_ic_-biotin was studied, the dose-response curves generated by RIA showed identity between TrxIA-2_ic_ and its biotinylated counterpart, according to the general principles of immunochemical cross reactivity [81]. Furthermore, competitive qualitative assays showed that all patient sera became virtually negative under TrxIA-2_ic_-biotin excess. All these results indicate that the recognition by IA-2A of both TrxIA-2_ic_ and the biotinylated protein was practically the same; therefore, it could be deduced that the biotinylation did not alter critical epitopes for interaction with IA-2A.

Stability studies in the storage condition at -20 °C in 100 mM 2ME lysis buffer indicated that as TrxIA-2_ic_ storage time proceeds, the displacing concentration required for a response half between B/B_0min_ and B/B_0max_ (IC_50_) was increasing to the point of no longer being recognized by IA-2A. However, TrxIA-2_ic_ immunoreactivity was maintained throughout the evaluation period when the fusion protein was kept at -20 °C in 50 % v/v glycerol, 50 mM 2ME lysis buffer. For this reason, the latter storage condition was selected, which ensured immunochemical quality of the recombinant antigen for at least four months, being applicable to routine immunoassays for the detection of IA-2A. This finding is one of the most interesting properties of the protein under study, as previous attempts in our laboratory failed to obtain IA-2_ic_ with sufficient stability to yield immunoassays for routine applications, as most of the soluble protein precipitated within 24 h.

Since TrxIA-2_ic_ could be expressed in a simple prokaryotic system with high yields, purity and stability, and it was properly identified and immunoreactive against IA-2A positive patient sera, we proceeded to apply this protein in non-radiometric methods for IA-2A detection. To start with, a population of 48 children and adolescents with newly diagnosed T1DM were subjected to IA-2A assessment by the reference method RBA, which shows the best sensitivity and specificity parameters in international quality controls (such as the Diabetes Autoantibody Standardization Program –DASP- and IASP). As previously described [64, 82], it is currently accepted that RBA alone is not enough to rule out the existence of autoimmunity in childhood diabetic patients; therefore, the performance of simultaneous determinations of all major markers (IAA, GADA, IA-2A and ZnT8A) is recommended. In this work, we managed to develop two methods capable of detecting IA-2A in some sera that RBA could not, probably due to differences in the principles of detection [83–85]. It has also been demonstrated that fluid phase radiometric assays may underestimate the IA-2A titers due to the formation of insoluble immune complexes which do not bind to Protein A-Sepharose, a phenomenon that would not occur in solid phase immunoassays [86].

The first alternative method, _bc_ELISA, included the development of a solid phase immunoassay, based on a standard design for detection of antibodies, similar to an indirect ELISA, characterized by the immobilization of the biotinylated antigen to the microplate via an avidin bridge [50, 61]. This design emerged as a viable option for solving the possible denaturation of the protein when adsorbed onto the plastic surface of the microplate [87], which would be deleterious for IA-2A binding mainly because they recognize discontinuous epitopes. Unfortunately, the performance parameters showed a narrow dynamic range and high signals with healthy control sera, with the consequent low sensitivity (39.6 %) and relative sensitivity (40.5 %) unsuitable for routine application. Another drawback of this assay is the requirement of a large sample volume, which makes it impractical when children and juvenile patients must be assessed. However, these performance values do not differ much from previous data corresponding to the same design for IA-2A [50, 59, 60] or GADA [61]. The poor performance could not be attributed to alterations in critical epitopes for recognition by IA-2A since it was shown that TrxIA-2_ic_-biotin exhibited immunoreactivity comparable to TrxIA-2_ic_. This phenomenon could be attributed to differences in the principles of antigen:antibody interaction (fluid phase vs. solid phase) and detection (radiometric vs. colorimetric).

In order to improve the performance in IA-2A assessment, we proceeded to the optimization of CL-_b_ELISA, the first described test for the determination of IA-2A with chemiluminescent detection. While this detection shows increased sensitivity, it can lead to higher non-specific signal as well. For this reason we decided to develop an immunoassay variant with high specificity. One way to reduce the background signal is to use the labelled specific antigen to detect bound antibody instead of the detection of total bound IgG with labelled xenogeneic anti-immunoglobulin antibody, which decreases the signal-to-background ratio [65]. Previous data obtained by our research group have shown that this design has acceptable performance values for the clinical application in routinely GADA determination in conventional ELISA or flow cytometry-based immunoassays [64, 82]. The design is based on the immobilization of the antigen to the solid phase, the incubation with the specific autoantibody (crosslinking molecule) and a fluid phase interaction with the biotinylated antigen via the available paratope. For detection of IA-2A, we demonstrated that it presented a wide dynamic range, even higher than that described for the reference method. The parameters of sensitivity (58.3 %), relative sensitivity (59.5 %) and specificity (94.9 %), although lower than those for RBA, are more than acceptable for use as a first line screening method for assessing humoral markers. It is difficult to compare the performance of this assay with those of previous studies where IA-2A is detected because in those studies different antigens were used, as well as different patient populations and healthy control individuals (with variables such as number, ethnicity and, possibly, sample matrix) [62, 63, 74–77]. The lower sensitivity showed by CL-_b_ELISA, in comparison with RBA, could be a consequence of partial denaturation of TrxIA-2_ic_ when adsorbed onto the surface of polystyrene microplates. Concerning the epitopes recognized by IA-2A, it was previously found that these autoantibodies mostly recognize the C-terminal domain of IA-2 [33]. Thus, it is not surprising that the Trx moiety, which is an N-terminal extension of Trx-IA-2_ic_, did not adversely affect the sensitivity of the ELISAs reported here. The lack of correlation between solid-phase and liquid-phase assays has been previously reported and discussed [84, 88–90]. In this sense, different thermodynamic principles apply to ELISA and RBA, since in the latter antigen–antibody reaction occurs in the liquid phase, in high dilution and near the equilibrium; meanwhile in both ELISA protocols the interaction occurs in solid phase, where the amount of immunocomplexes is highly dependent on antibodies concentration. In fact, in T1DM, autoantibodies concentration is really low (in the order of 10^-12^ M), turning their detection into an analytical challenge using solid phase assays, especially for sensitivity limitations. Then, as expected, there was a low correlation between the results obtained by both ELISA protocols and RBA. This protocol also used a lower volume of sera than _bc_ELISA, but still higher than that used in RBA (2.5 μL of undiluted sera). Concerning healthy control individuals, the presence of possible false positive may be due to a matrix effect caused by samples from patients who did not have the proper fasting as diabetic patients did. As mentioned above, the presence of anti-Trx crossreactivity in healthy control sera could be ruled out [53]. When a comparison was made with _bc_ELISA, CL-_b_ELISA had higher values for the above mentioned analytical parameters. In addition, the differences in dynamic range between methods can be explained, at least partially, by higher sensitivity with chemiluminiscent detection and increased signal-to-background ratio in immunoassay design. As regards differential detection of immunoglobulin isotypes, while CL-_b_ELISA can potentially detect any, other immunoassays have limitations for binding specificity to the antibody conjugated to peroxidase (_bc_ELISA) or protein A (RBA). In order to better characterize the performance of CL-_b_ELISA, we increased the number of type 1 diabetic patients (*n* = 60) and healthy controls (*n* = 115) samples already tested (Additional file 6: Figure S6). As a result, a sensitivity of 56.7 %, an analytical sensitivity of 59.6 % and a specificity of 97.4 % were obtained. It is noteworthy that in the DASP and the IASP the number of samples that are received to evaluate the analytical performance of different immunoassays is similar to the number used in this work. Employing a higher number of samples, the sensitivity values remained fairly the same; however a slightly increased in the specificity of the assay was observed since the percentage of detected outliers decreased in a more representative population. This data reinforces the applicability of CL-_b_ELISA in IA-2A routine determination.

## Conclusion

The findings of this study can be summarized in the following statements: i) the recombinant vector pTrxIA-2_ic_ was designed, synthesized and quality-controlled, ii) soluble human IA-2_ic_ was expressed as a properly folded thioredoxin fusion protein in *E. coli*, iii) higher levels of TrxIA-2_ic_ expression were obtained after 3 h of induction on GI724 strain, iv) highly pure TrxIA-2_ic_ was recovered from the intracellular soluble fraction after a single step purification by affinity chromatography, v) the MW of TrxIA-2_ic_ could be estimated by SDS-PAGE, SEC and MS, vi) the presence of TrxIA-2_ic_ soluble monomers and covalent-mediated dimers was determined under native conditions, vii) TrxIA-2_ic_ was properly identified by WB and MS, viii) excellent immunochemical behavior of properly folded full TrxIA-2_ic_ was validated by inhibition or displacement of [^35^S]IA-2 binding from IA-2A, ix) great stability over time was found under proper storage conditions and x) low cost and environmentally harmless non-radiometric methods for IA-2A assessment were developed, which are accessible to many laboratories.

Furthermore, methods for IA-2A detection by flow cytometry based on the use of polystyrene microspheres, with the potential simultaneous determination of the main humoral markers of autoimmune DM are currently being developed in our laboratory [64], since multiplex testing will facilitate high-throughput screening of T1DM in the general population [91]. It is clear that better tests based on pure antigen preparations will allow better screening of DM risk, characterizing the course of the disease and the distinction between DM of immune origin and that of metabolic cause.

## Additional files

Additional file 1: Figure S1. Affinity purification of thioredoxin fusion proteins with an agarose-based support covalently modified with phenylarsine oxide. Thioredoxin fusion proteins, containing vicinal dithiols in their active site (CysGlyProCys region), reversibly bind the hydrophobic trivalent arsenic functional site and are eluted using 2-mercaptoethanol (2ME). Affi-Gel 10 (Bio-Rad Laboratories Inc.) was used as the agarose-based support. (PDF 170 kb)
Additional file 2: Figure S2. TrxIA-2_ic_ identification by western blot in eluted fractions from size exclusion chromatography and dithiothreitol reduction analysis. WB was revealed with a rabbit polyclonal serum to IA-2_ic_ as primary antibody. A: Identification of monomeric and dimeric forms of TrxIA-2_ic_. Lane 1: affinity purified TrxIA-2_ic_from *E. coli* GI724 strain; lane 2: eluted fraction at V_e_ = 8.75 mL, corresponding to dimeric TrxIA-2_ic_; lane 3: eluted fraction at V_e_ = 9.79 mL, corresponding to monomeric TrxIA-2_ic_; B: Conversion to TrxIA-2_ic_ monomeric form under dithiothreitol reduction. Lane 1: TrxIA-2_ic_ under denaturalizing and reducing conditions (8 M urea and 100 mM dithiothreitol). Arrows indicate the electrophoretic mobility of monomeric TrxIA‑2_ic_. (PDF 186 kb)
Additional file 3: Figure S3. Mass spectrometric analysis of TrxIA-2_ic_. A molecular weight of 55,209.008 Da indicates the major peak consistent with the expected molecular mass of TrxIA-2_ic_. Samples were analyzed on a Bruker Microflex MALDI-TOF device. (PDF 204 kb)
Additional file 4: Figure S4. Competitive quantitative assay for TrxIA-2_ic_ and TrxIA-2_ic_-biotin synthesized with high rates of biotinylation. Dose-response curves for a pool of 3 IA-2A RBA positive Type 1 diabetic patient sera for different concentrations of TrxIA-2_ic_ (open circle, solid line) and TrxIA-2_ic_-biotin (closed circle, dotted line). Parallelism and identity between curves was achieved (one curve adequately fits all data, alpha = 0.05, *R* ^2^ = 0.9653). (PDF 111 kb)
Additional file 5: Figure S5. IA-2_ic_ expression as a fusion protein with Trx in *E. coli* GI724 inclusion bodies. A: SDS-PAGE (12.1 % T 6.0 % C, 1 mm, under reducing conditions, stained with Coomassie Brilliant Blue R-250), B: WB revealed with a rabbit polyclonal serum to thioredoxin as primary antibody. Lanes 1–4: samples from pTrxIA-2_ic_ transformed *E. coli* GI724 strain. Lane 1: sample before induction (0 h); lane 2: sample after 1.5 h of induction; lane 3: sample after 3.0 h of induction; lane 4: sample after 16.0 h of induction. Arrows indicate the electrophoretic mobility of TrxIA-2_ic_. (PDF 254 kb)
Additional file 6: Figure S6. CL-_b_ELISA for IA-2A assessment in 115 healthy control and 60 Type 1 diabetic patient sera. The results are expressed as SDs, the cut-off value (2.5) is indicated by a dotted line and medians for each population are indicated by a dashed line. Out of the 60 patient sera, 34 scored positive (56.7 % sensitivity), whereas 26 patients were negative. Thirty one IA-2A positive sera by RBA scored positive when evaluated by CL-_b_ELISA, showing an analytical sensitivity of 59.6 %, SDs ranging from -1.010 to 111.20 and a median SDs = 3.18. Healthy control sera (*n* = 115) results showed a specificity of 97.4 %. (PDF 237 kb)

## Abbreviations

2ME: 2-mercaptoethanol
_bc_ELISA: Blank corrected ELISA with colorimetric detection
CL-_b_ELISA: Bridge ELISA with chemiluminescent detection
cps: Counts per second
CV: Column volume
DASP: Diabetes Autoantibody Standardization Program
DM: Diabetes Mellitus
DSG: Dense core secretory granules
EK: Enterokinase
GAD: Glutamic acid decarboxylase
GADA: Glutamic acid decarboxylase autoantibodies
HRP: Horseradish Peroxidase
IA-2: Insulinoma associated protein tyrosine phosphatase 2
IA-2A: Insulinoma associated protein tyrosine phosphatase 2 autoantibodies
IA-2_ic_His_6_: IA-2_ic_ fused to a C-terminal His-tag
IAA: Insulin autoantibodies
IASP: Islet Autoantibody Standardization Program
IB: Inclusion bodies
ISF: Intracellular soluble fraction
K_a_: Affinity constant
LADA: Latent autoimmune diabetes of the adults
MS: Mass spectrometry
MW: Molecular weight
ON: Overnight
PBS: Phosphate-buffered saline
PBS-M: 2 % w/v skim milk in PBS
PBS-MT: 2 % w/v skim milk, 0.05 % v/v Tween 20 in PBS
PBS-T: PBS containing 0.05 % v/v Tween 20
PTP: Protein tyrosine phosphatase
RBA: Radioligand binding assay
RIA: Radioimmunoassay
RT: Room temperature
SDs: Standard deviation scores
SDS-PAGE: Sodium dodecyl sulphate-polyacrylamide gel electrophoresis
SEC: Size-exclusion chromatography
T1DM: Type 1 Diabetes mellitus
TBS-MT: 3 % w/v skim milk, 0.05 % v/v Tween 20 in TBS
TBS-T: 0.05 % v/v Tween 20 in TBS
TCL: Total cell lysate
Trx: Thioredoxin
TrxIA-2_ic_: Intracellular domain of IA-2 fused to thioredoxin
V_e_: Elution volume
WB: Western blot
ZnT8: Zinc transporter 8
ZnT8A: Zinc transporter 8 autoantibodies
