# Supplementary material for: Novel prokaryotic expression of thioredoxin-fused insulinoma associated protein tyrosine phosphatase 2 (IA-2), its characterization and immunodiagnostic application
Source: BMC Biotechnol. 2016 Nov 24;16:84. doi: 10.1186/s12896-016-0309-2 (PMC5122161; doi:10.1186/s12896-016-0309-2)
Supplement: Additional file 1: Figure S1. — Affinity purification of thioredoxin fusion proteins with an agarose-based support covalently modified with phenylarsine oxide. Thioredoxin fusion proteins, containing vicinal dithiols in their active site (CysGlyProCys region), reversibly bind the hydrophobic trivalent arsenic functional site and are eluted using 2-mercaptoethanol (2ME). Affi-Gel 10 (Bio-Rad Laboratories Inc.) was used as the agarose-based support. (PDF 170 kb) [file 12896_2016_309_MOESM1_ESM.pdf]

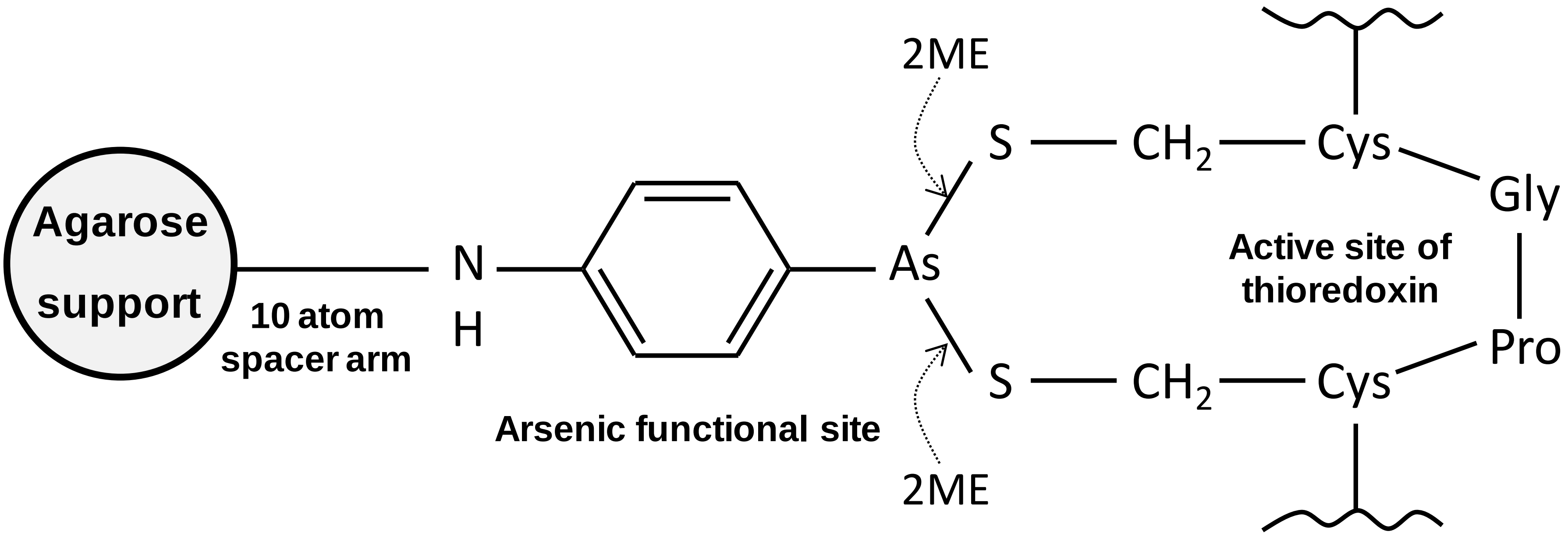

Figure S1. Affinity purification of thioredoxin fusion proteins with an agarose-based support covalently modified with phenylarsine oxide.
