# Supplementary material for: Novel prokaryotic expression of thioredoxin-fused insulinoma associated protein tyrosine phosphatase 2 (IA-2), its characterization and immunodiagnostic application
Source: BMC Biotechnol. 2016 Nov 24;16:84. doi: 10.1186/s12896-016-0309-2 (PMC5122161; doi:10.1186/s12896-016-0309-2)
Supplement: Additional file 2: Figure S2. — TrxIA-2ic identification by western blot in eluted fractions from size exclusion chromatography and dithiothreitol reduction analysis. WB was revealed with a rabbit polyclonal serum to IA-2ic as primary antibody. A: Identification of monomeric and dimeric forms of TrxIA-2ic. Lane 1: affinity purified TrxIA-2icfrom E. coli GI724 strain; lane 2: eluted fraction at Ve = 8.75 mL, corresponding to dimeric TrxIA-2ic; lane 3: eluted fraction at Ve = 9.79 mL, corresponding to monomeric TrxIA-2ic; B: Conversion to TrxIA-2ic monomeric form under dithiothreitol reduction. Lane 1: TrxIA-2ic under denaturalizing and reducing conditions (8 M urea and 100 mM dithiothreitol). Arrows indicate the electrophoretic mobility of monomeric TrxIA‑2ic. (PDF 186 kb) [file 12896_2016_309_MOESM2_ESM.pdf]

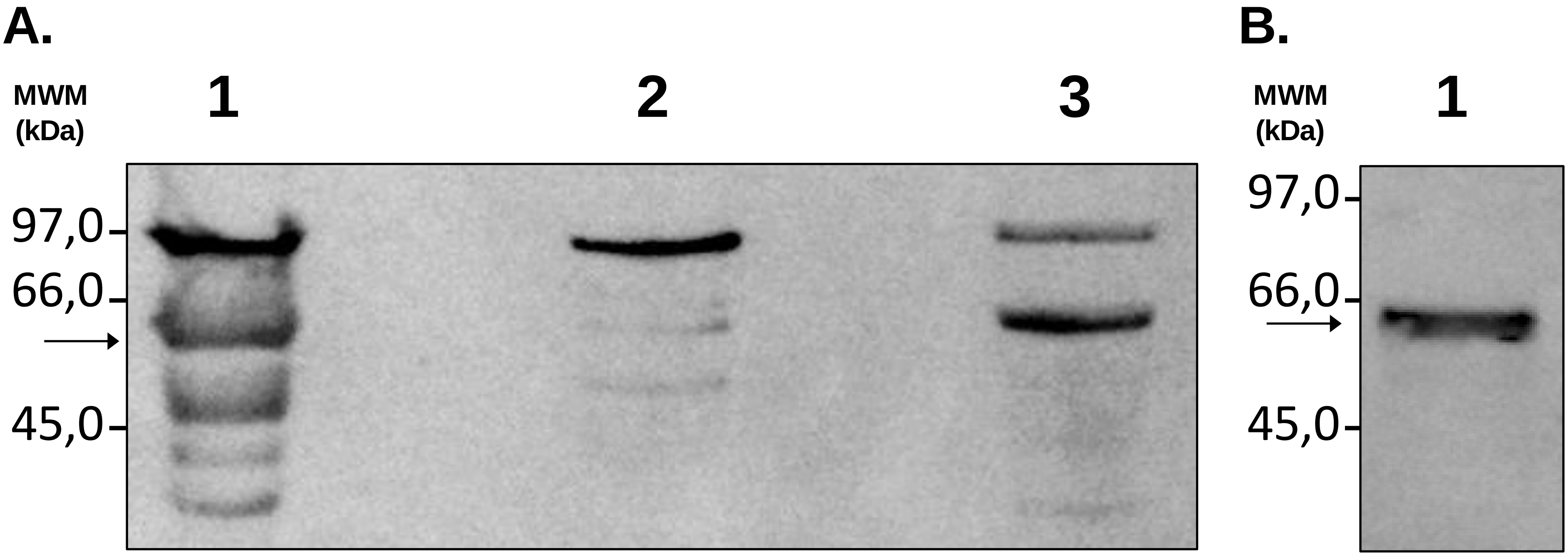

Figure S2. TrxIA-2_{ic} identification by western blot in eluted fractions from size exclusion chromatography and dithiothreitol reduction analysis.
