# Supplementary figures and images for: Novel prokaryotic expression of thioredoxin-fused insulinoma associated protein tyrosine phosphatase 2 (IA-2), its characterization and immunodiagnostic application
Source: BMC Biotechnol. 2016 Nov 24;16:84. doi: 10.1186/s12896-016-0309-2 (PMC5122161; doi:10.1186/s12896-016-0309-2)

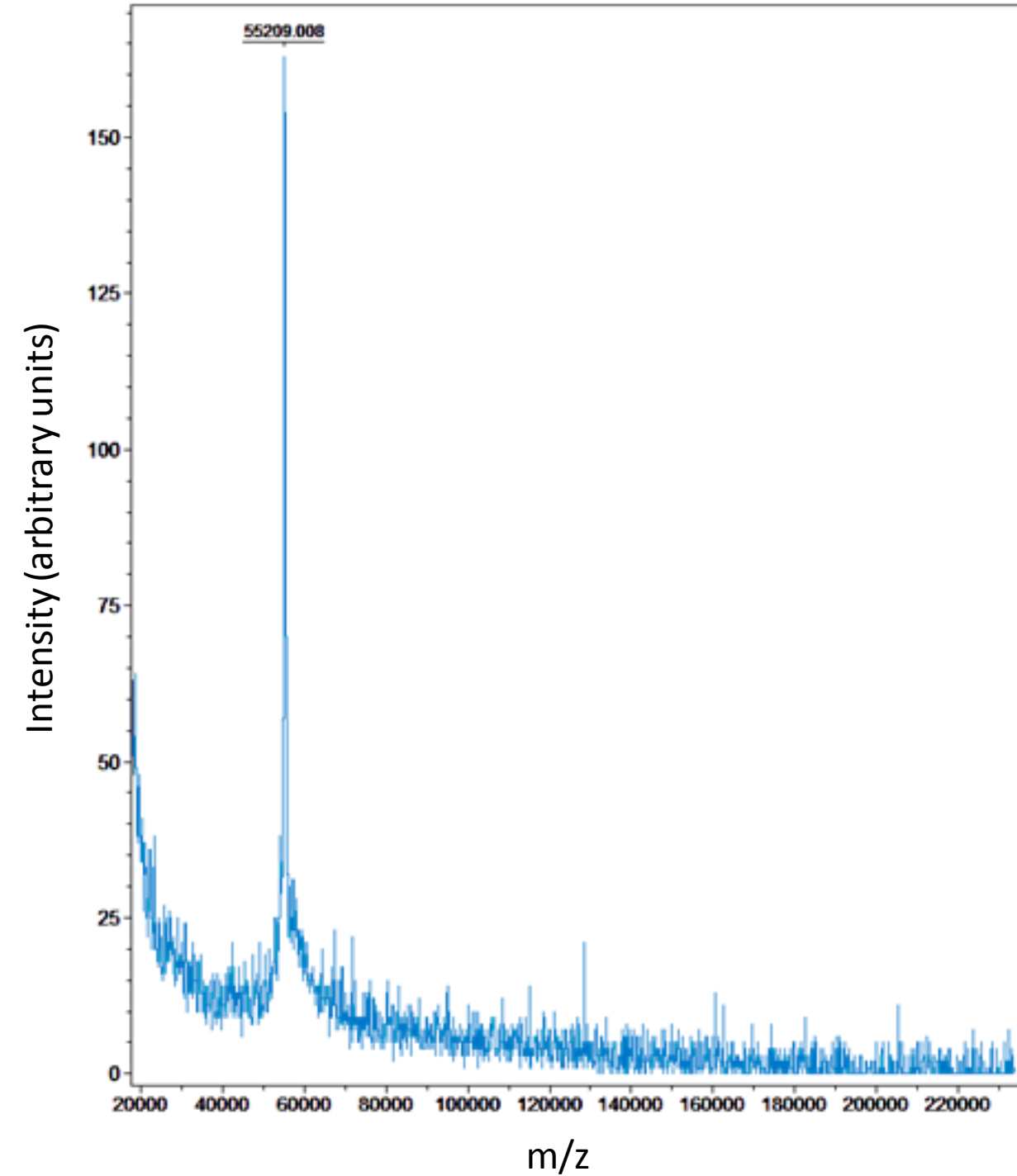

**Figure S3. Mass spectrometric analysis of TrxIA-2<sub>ic</sub>.**

Supplement: Additional file 3: Figure S3. — Mass spectrometric analysis of TrxIA-2ic. A molecular weight of 55,209.008 Da indicates the major peak consistent with the expected molecular mass of TrxIA-2ic. Samples were analyzed on a Bruker Microflex MALDI-TOF device. (PDF 204 kb) [file 12896_2016_309_MOESM3_ESM.pdf]

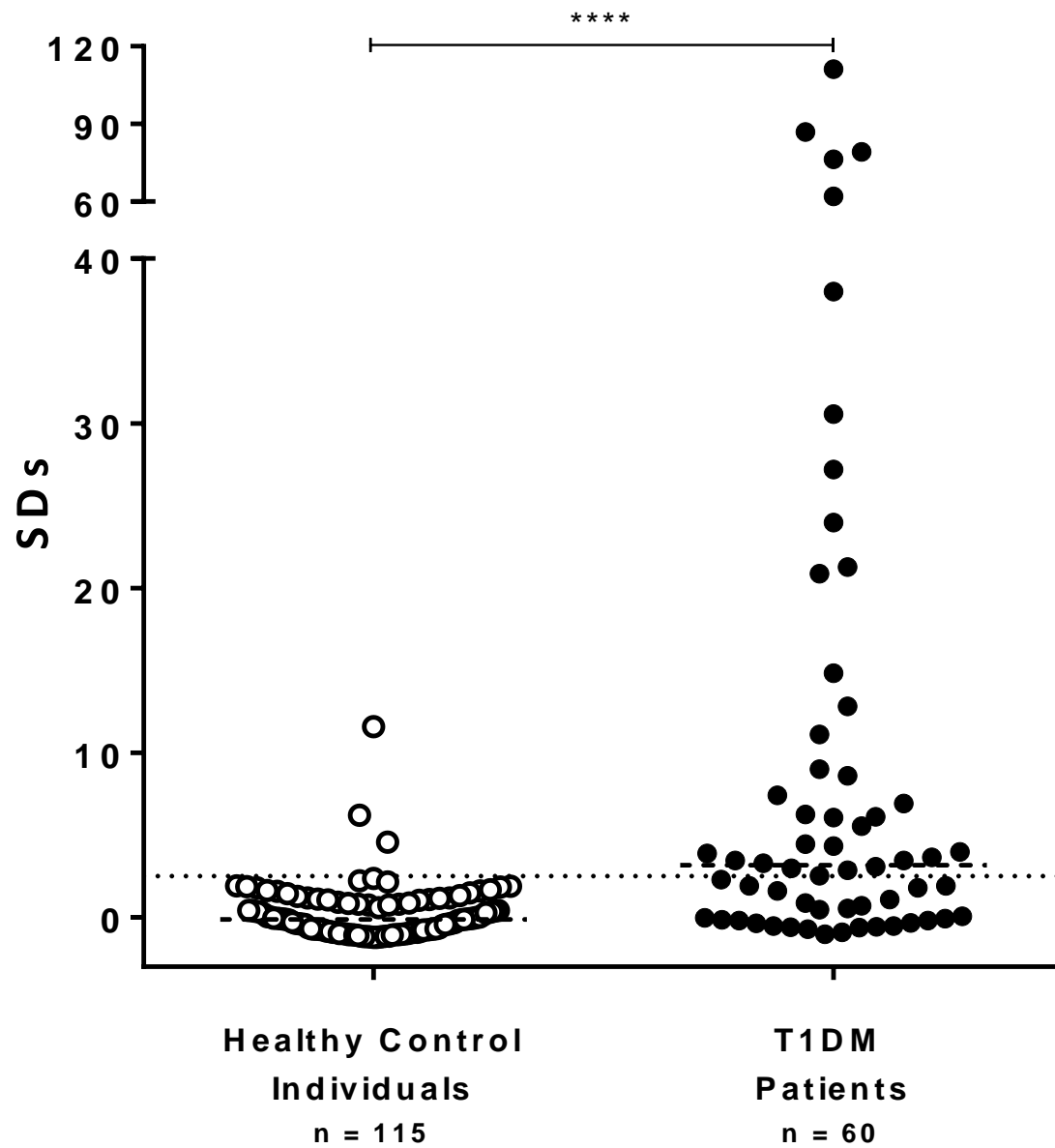

**Figure S6. CL-bELISA for IA-2A assessment in 115 healthy control and 60 Type 1 diabetic patient sera.**

Supplement: Additional file 6: Figure S6. — CL-bELISA for IA-2A assessment in 115 healthy control and 60 Type 1 diabetic patient sera. The results are expressed as SDs, the cut-off value (2.5) is indicated by a dotted line and medians for each population are indicated by a dashed line. Out of the 60 patient sera, 34 scored positive (56.7 % sensitivity), whereas 26 patients were negative. Thirty one IA-2A positive sera by RBA scored positive when evaluated by CL-bELISA, showing an analytical sensitivity of 59.6 %, SDs ranging from -1.010 to 111.20 and a median SDs = 3.18. Healthy control sera (n = 115) results showed a specificity of 97.4 %. (PDF 237 kb) [file 12896_2016_309_MOESM6_ESM.pdf]
