# Supplementary material for: Novel prokaryotic expression of thioredoxin-fused insulinoma associated protein tyrosine phosphatase 2 (IA-2), its characterization and immunodiagnostic application
Source: BMC Biotechnol. 2016 Nov 24;16:84. doi: 10.1186/s12896-016-0309-2 (PMC5122161; doi:10.1186/s12896-016-0309-2)

Figure S4. Competitive quantitative assay for TrxIA-2_{ic} and TrxIA-2_{ic}-biotin synthesized with high rates of biotinylation.

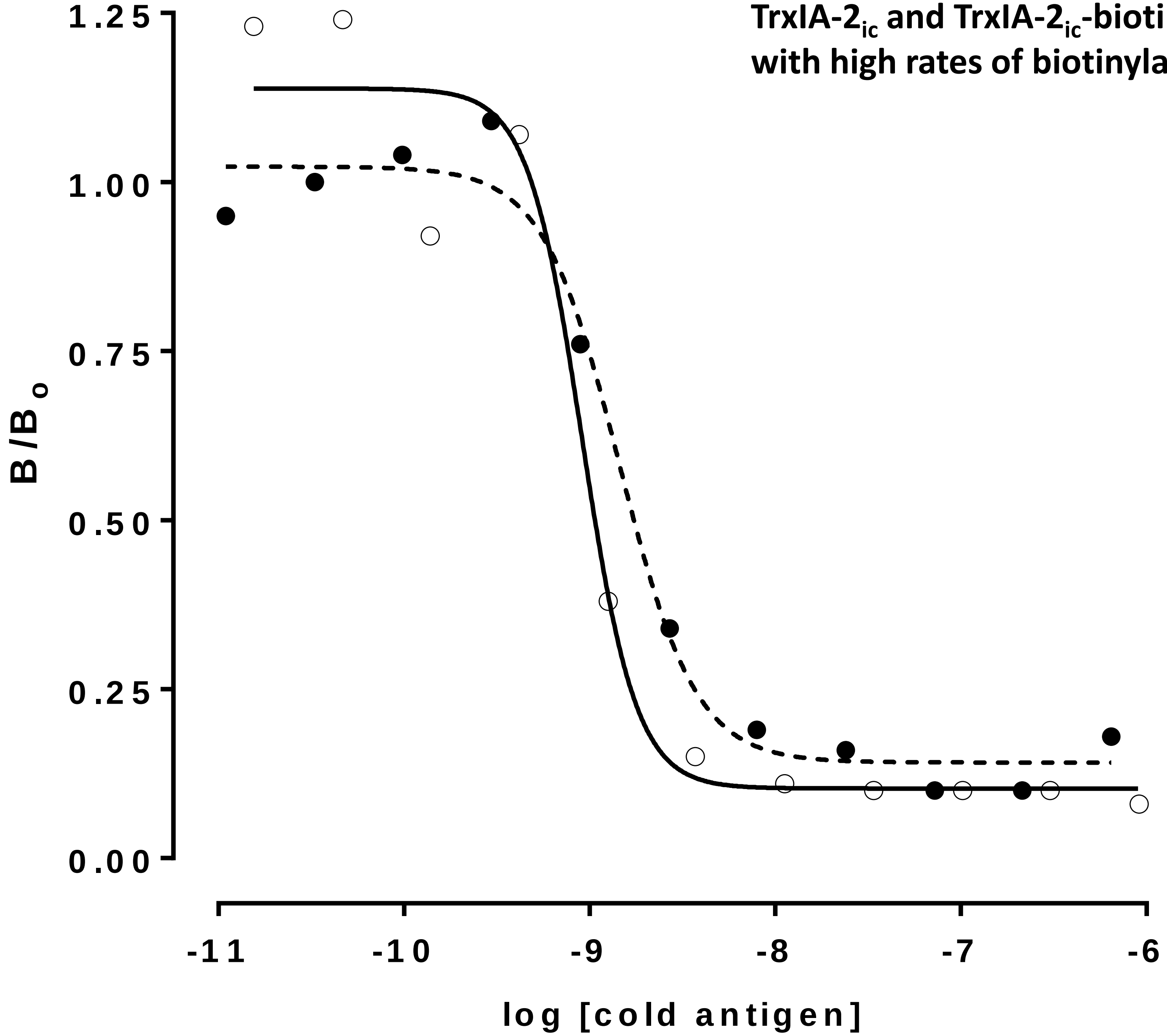

Supplement: Additional file 4: Figure S4. — Competitive quantitative assay for TrxIA-2ic and TrxIA-2ic-biotin synthesized with high rates of biotinylation. Dose-response curves for a pool of 3 IA-2A RBA positive Type 1 diabetic patient sera for different concentrations of TrxIA-2ic (open circle, solid line) and TrxIA-2ic-biotin (closed circle, dotted line). Parallelism and identity between curves was achieved (one curve adequately fits all data, alpha = 0.05, R 2 = 0.9653). (PDF 111 kb) [file 12896_2016_309_MOESM4_ESM.pdf]
