# Supplementary material for: Novel prokaryotic expression of thioredoxin-fused insulinoma associated protein tyrosine phosphatase 2 (IA-2), its characterization and immunodiagnostic application
Source: BMC Biotechnol. 2016 Nov 24;16:84. doi: 10.1186/s12896-016-0309-2 (PMC5122161; doi:10.1186/s12896-016-0309-2)
Supplement: Additional file 5: Figure S5. — IA-2ic expression as a fusion protein with Trx in E. coli GI724 inclusion bodies. A: SDS-PAGE (12.1 % T 6.0 % C, 1 mm, under reducing conditions, stained with Coomassie Brilliant Blue R-250), B: WB revealed with a rabbit polyclonal serum to thioredoxin as primary antibody. Lanes 1–4: samples from pTrxIA-2ic transformed E. coli GI724 strain. Lane 1: sample before induction (0 h); lane 2: sample after 1.5 h of induction; lane 3: sample after 3.0 h of induction; lane 4: sample after 16.0 h of induction. Arrows indicate the electrophoretic mobility of TrxIA-2ic. (PDF 254 kb) [file 12896_2016_309_MOESM5_ESM.pdf]

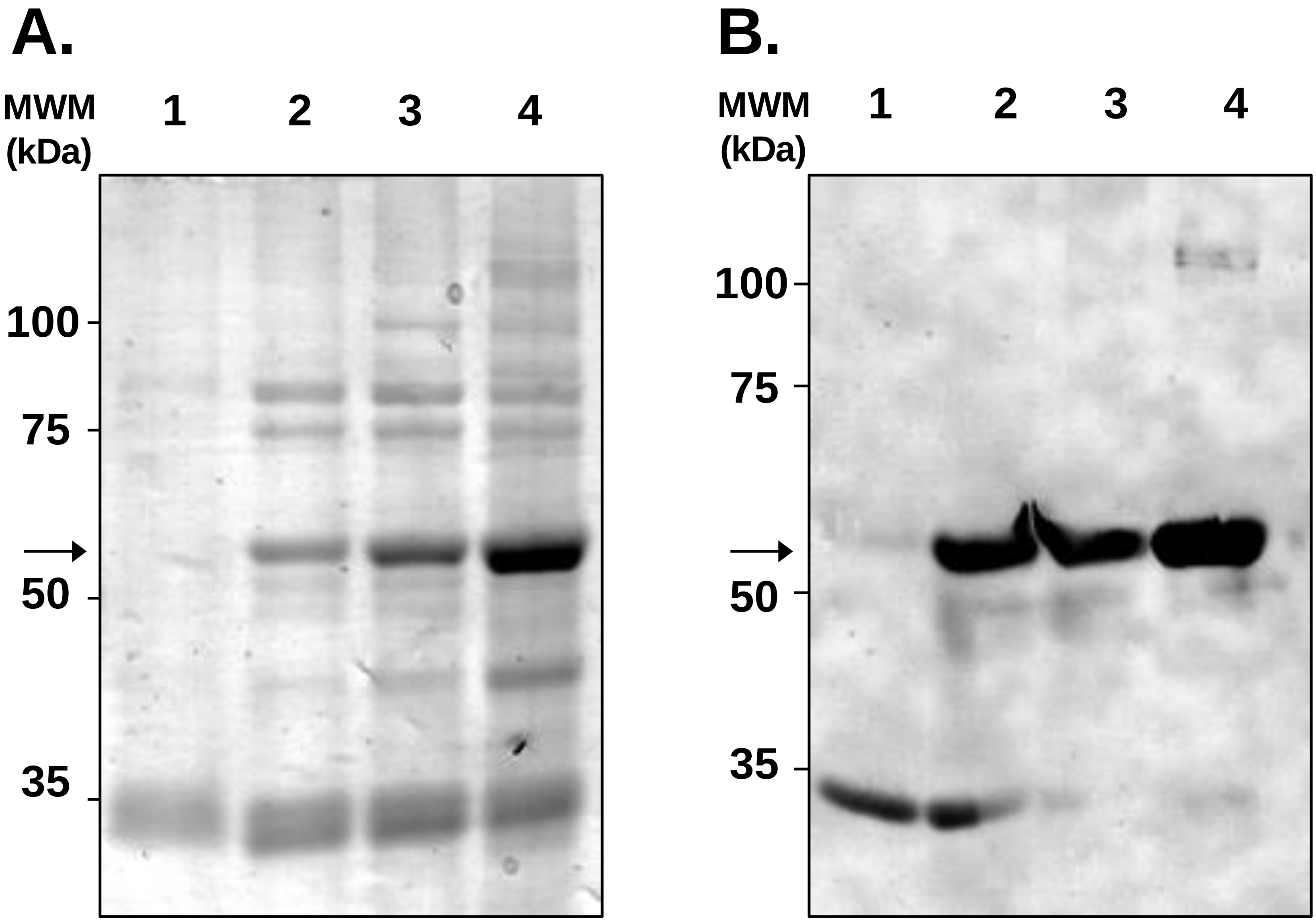

Figure S5. IA-2$_{ic}$ expression as a fusion protein with Trx in *E. coli* GI724 inclusion bodies.
